# Engineering of α-PD-1 antibody-expressing long-lived plasma cells by CRISPR/Cas9-mediated targeted gene integration

**DOI:** 10.1038/s41419-020-03187-1

**Published:** 2020-11-12

**Authors:** Baohong Luo, Yikang Zhan, Minqi Luo, Huimin Dong, Jun Liu, Yingtong Lin, Junsong Zhang, Guanwen Wang, Els Verhoeyen, Yiwen Zhang, Hui Zhang

**Affiliations:** 1 Institute of Human Virology, Zhongshan School of Medicine, Sun Yat-sen University, 510080 Guangzhou, Guangdong China; 2 Key Laboratory of Tropical Disease Control of Ministry of Education, Zhongshan School of Medicine, Sun Yat-sen University, 510080 Guangzhou, Guangdong China; 3 Guangdong Engineering Research Center for Antimicrobial Agent and Immunotechnology, Zhongshan School of Medicine, Sun Yat-sen University, 510080 Guangzhou, Guangdong China; 4 Department of Laboratory Medicine, Third Affiliated Hospital of Sun Yat-sen University, 510080 Guangzhou, Guangdong China; 5 CIRI – International Center for Infectiology, Research team EVIR, Inserm, U1111, CNRS, UMR5308, Ecole Normale Supérieure de Lyon, Université Claude Bernard Lyon 1, University of Lyon, Lyon, France; 6 Université Côte d’Azur, INSERM, C3M, 06204 Nice, France

**Keywords:** Cancer immunotherapy, B cells

## Abstract

Long-lived plasma cells (LLPCs) are robust specialized antibody-secreting cells that mainly stay in the bone marrow and can persist a lifetime. As they can be generated by inducing the differentiation of B-lymphocytes, we investigated the possibility that human LLPCs might be engineered to express α-PD-1 monoclonal antibody to substitute recombinant α-PD-1 antitumor immunotherapy. To this end, we inserted an α-PD-1 cassette into the *GAPDH* locus through Cas9/sgRNA-guided specific integration in B-lymphocytes, which was mediated by an integrase-defective lentiviral vector. The edited B cells were capable of differentiating into LLPCs both in vitro and in vivo. Transcriptional profiling analysis confirmed that these cells were typical LLPCs. Importantly, these cells secreted de novo antibodies persistently, which were able to inhibit human melanoma growth via an antibody-mediated checkpoint blockade in xenograft-tumor mice. Our work suggests that the engineered LLPCs may be utilized as a vehicle to constantly produce special antibodies for long-term cellular immunotherapy to eradicate tumors and cellular reservoirs for various pathogens including human immunodeficiency virus type 1 (HIV-1) and hepatitis B virus (HBV).

## Introduction

B-lymphocytes are a special class of immune cells that provide specific immune surveillance mainly by producing various antibodies^1,2^. The downstream effectors, plasmablasts (PBs) and plasma cells, are specialized antibody-secreting cells^3^. Under physiological conditions, B-lymphocytes differentiate into short-lived PBs in the germinal centers of lymph nodes and the spleen, and subsequently travel to the bone marrow, where they receive survival signals from special niches and differentiate into long-lived plasma cells (LLPCs)^4,5^. LLPCs may persist a lifetime and maintain a continuous supply of serum antibodies^4,5^. It has been demonstrated that human primary B cells and plasma cells can be engineered to produce therapeutic antibodies and proteins, such as anti-HCV and anti-HIV broadly neutralizing antibodies (bnAbs) and human factor IX (FIX)^6–10^. Since strategies for reprogramming primary B cells and plasma cells have been developed, it is reasonable to hypothesize that LLPCs could be explored as a novel platform for long-term gene therapeutics.

Delivery tool efficiency and safety are of major concern in genetically engineering of human primary B cells and plasma cells. The bacteria-originated clustered regularly interspaced short palindromic repeats (CRISPR) associated protein 9 (Cas9) system, which allows for a highly efficient modification at specific genetic loci in primary human cells^11,12^, has been used as a tool to engineer B-lymphocytes^13–20^. The CRISPR-Cas9–mediated permanent genome editing in B-lymphocytes decreases the risk of mutagenesis from random insertion and is superior to the short-term expression by viral vector transduction in engineered B-lymphocyte therapeutics. However, it is important to choose a reasonable delivery system for the CRISPR-Cas9 to edit B cells. Because the recombinant adeno-associated virus (AAV) vector does not integrate and allows for persistent expression, it has been chosen as the delivery system for B-cell editing^16,17,20^. However, the AAV vector has limited packaging capacity (~4.7 kb) and its transduction efficiency into B-cells is unsatisfactory^16^.

Conversely, integrase-defective lentiviral vector (IDLV), which carries a mutated form of integrase, is defective for integration into host chromosomes while being sufficiently competent for the transduction and nuclear delivery of nonintegrative forms of vector DNA^21,22^. IDLV exhibits the advantages associated with lentiviral vector (LV), including a larger packaging capacity (~10 kb), efficient transduction, low cytotoxicity, and immunogenicity, but does not exhibit the disadvantages of LVs such as random integration into the genome^21,23^. Due to these characteristics, IDLV is an ideal vehicle to ensure transient expression of the CRISPR/Cas9 system. It has already been used as a vehicle for delivering CRISPR/Cas9 safely and efficiently into various cells^24,25^. Importantly, Hoban et al.^26^ used this transient delivery of CRISPR/Cas9 reagents to successfully target and modify CD34^+^ hematopoietic stem cells. However, it remains to be determined whether IDLVs could function as a Cas9 delivery tool to edit human primary B cells and plasma cells.

Programmed death 1 (PD-1), one of the immune checkpoint molecules, is mainly expressed in activated T cells^27–29^. Along with its ligand programmed death ligand 1 (PD-L1), PD-1/PD-L1 are the central regulators of T cell exhaustion^30,31^. Immune checkpoint blockades, mediated by PD-1 blocking monoclonal antibodies (mAbs) such as pembrolizumab and nivolumab, have shown remarkable effects for treatment of advanced melanomas in clinical practice^32–35^. However, the optimal duration of α-PD-1 therapy remains to be established. In most clinical trials, patients were able to continue α-PD-1 therapy until the development of a progressive disease, a treatment-related toxicity, or a maximum treatment time of 2 years^32–35^. Recently, a prospective study demonstrated that retreatment with α-PD-1 therapy led to renewed antitumor activity after a treatment break^36^. Since immune checkpoint blockade therapy requires continuous administration of mAbs by repeated injections, we hypothesized that adoptive B-lymphocytes or LLPC-based immunotherapy for long-term transgenic antibody expression may potentially substitute the repeated injections of α-PD-1 mAb.

Our current study developed a convenient procedure for delivering Cas9/sgRNA and a corresponding donor template into human primary B cells by using an IDLV delivery system. As a result, the α-PD-1 cassette was integrated into the *GAPDH* locus with high efficiency. Furthermore, these gene-edited B cells could differentiate into LLPCs, both in vitro and in vivo in humanized mice, which were responsible for maintaining of serum antibody titers for a long time. We have also shown that α-PD-1 mAb produced by these genetically engineered LLPCs exhibit effective antitumor effects in melanoma-inoculated mice.

## Results

### CRISPR-Cas9-mediated targeted transgene insertion by IDLV delivery

**Fig. 1:**
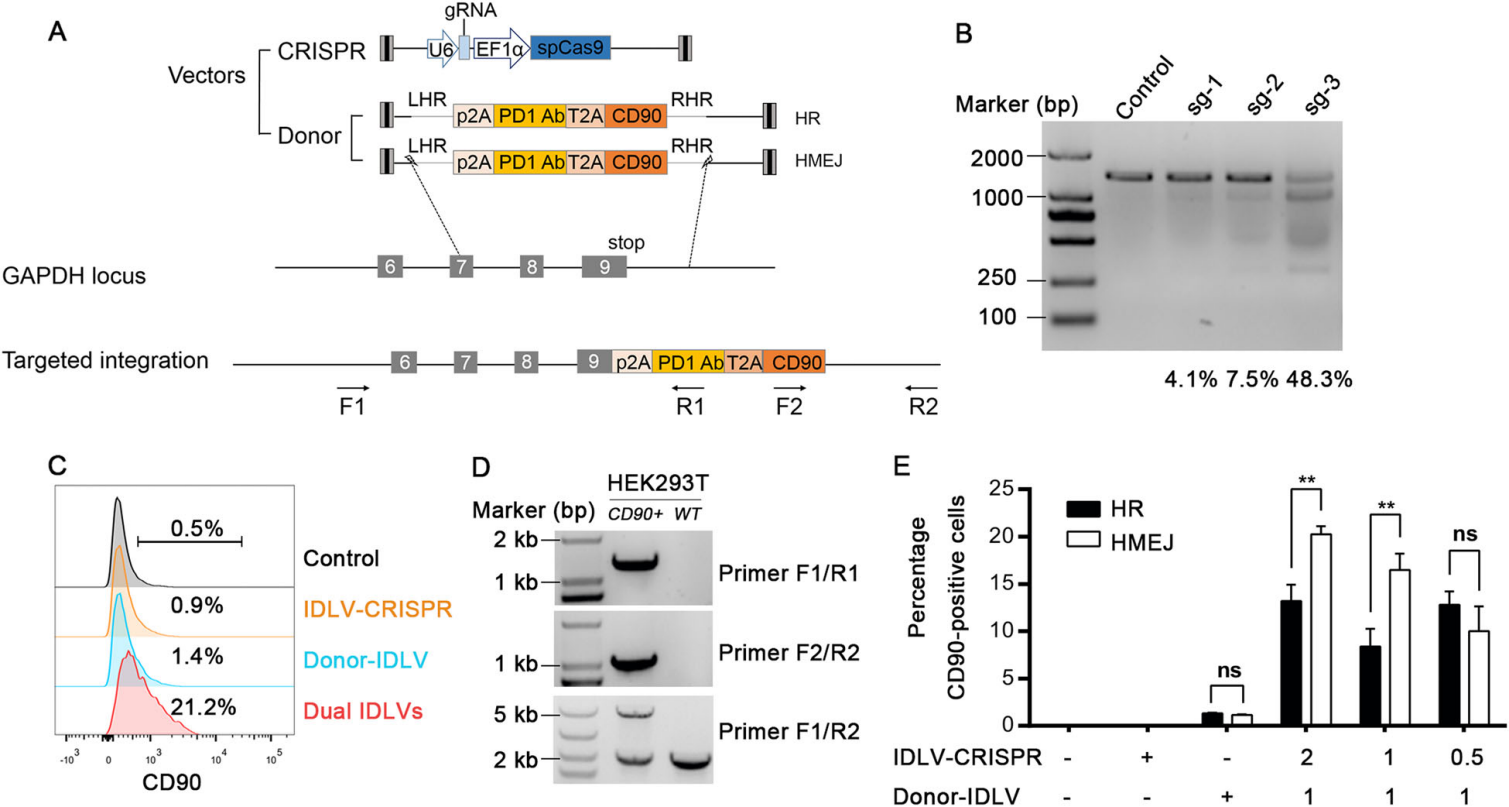
CRISPR-Cas9-mediated targeted integration of the α-PD-1 cassette into the *GAPDH* locus in HEK293T cells via IDLV delivery. **a** Schematic overview of the donor plasmid, Cas9/sgRNA expression plasmid, and targeting strategy for α-PD-1 integration into *GAPDH* 3′-UTR. Positions of the PCR primers (black arrows) used for detection of integrated DNA fragments are indicated. Fine gray lines on donor plasmids indicate sections homologous to the *GAPDH* locus. Lightning shape, sgRNA target sequence, HR, homologous recombination-based strategy, HMEJ, homology-mediated end joining-based strategy, LHR/RHR, left/right arm of homology recombination, F1/R2, outer forward/reverse primer, F2/R1, inner forward/reverse primer. **b** The mismatch-sensitive endonuclease T7E1 assay results showed the different efficiencies of Cas9/sgRNA-1, 2, and 3 for targeting human HEK293T genome. HEK293T cells were transfected with Cas9/sgRNA-1, 2 or 3 expression plasmid, without donor plasmid. Genomic DNA was extracted for T7E1 assay at day 4 post transfection. **c** FACS analysis of HEK293T cells showed the knock-in efficiencies of the α-PD-1 mAb in HEK293T cells. IDLV with HR-donor alone, IDLV expressing Cas9/sgRNA alone, or the two IDLVs together, were allowed to infect HEK293T cells. Control without IDLV infection is shown on the top. **d** CD90^+^ cells were sorted for genomic PCR analysis. Two sets of primers specific for the 5′ or 3′ integration junctions were used. Primer pair F1/R1 and F2/R2 amplified the 5′-junction (1435 bp) and the 3′-junction (1008 bp) of the transgene integration respectively. Primers F1/R2 amplified two DNA fragments that represent the wild type (2176 bp) and modified gene (4929 bp), respectively. **e** Relative knock-in efficiencies of HR and HMEJ-based strategies in HEK293T cells. Cells were infected with IDLV carried HR-donor or HMEJ-donor along with IDLV expressing Cas9/sgRNA at different MOIs. CD90 expression was analyzed by FACS 5 days post infection. Data are representative of three independent experiments (means ± SEM), **P < 0.01, ns, no significant difference; two-tailed Student’s *t* test (**e**) was used.

In order to efficiently mediate the targeted integration of the α-PD-1 transgene into the locus of the housekeeping gene *GAPDH* and ensure persistent gene expression, we generated a donor plasmid to carry a promoterless P2A-α-PD-1 sequence flanked by two *GAPDH* homology arms (HAs), named homologous recombination (HR) donor. Of note, homology-mediated end joining (HMEJ)-based strategy could improve the efficiency of homology-mediated gene integration^37^. We therefore constructed an HMEJ donor containing the guide RNA target sites on either side of the HAs (Fig. 1a). To conveniently evaluate the knock-in efficiency, we fused a T2A-CD90 reporter gene downstream of the α-PD-1 gene to allow the co-expression of CD90 on the cell surface, which was similar to α-PD-1 being under the control of the *GAPDH* promoter, and thus functioned as a convenient marker for evaluating the insertion efficiency (Fig. 1a).

To determine an appropriate position that favors transgene expression and allows simultaneous expression of the endogenous *GAPDH* gene, we designed three sgRNAs to target the *GAPDH* 3ʹ-UTR in close proximity to the stop codon of the coding sequence (CDS). Based on results of the T7 endonuclease I (T7E1) assay^38^, we selected sgRNA3, which produced 48.3% indels, as the guiding target site (Fig. 1b). Next, to assess HR efficiency, IDLV with HR-donor alone, IDLV expressing Cas9/sgRNA alone, or the two IDLVs together, were allowed to infect HEK293T cells. The HR efficiency, evidenced by CD90 expression on the cellular surface, was quantitatively analyzed via flow cytometry. We detected >20% CD90-positive cells in the experimental group receiving two IDLVs (Fig. 1c). The targeted site-specific integration was further confirmed by PCR on the genomic DNA and sequencing analysis (Fig. 1d). Lastly, we compared the efficiency of the HR-based method with that of the HMEJ-based method using increasing doses of the IDLVs expressing Cas9/sgRNA. We found that the HMEJ-based method exhibited higher knock-in efficiency with the recombinant viruses (Fig. 1e). Overall, these experiments showed that co-delivery of donor- and Cas9/sgRNA-IDLVs resulted in effective and specific expression of the transgene after integration into the *GAPDH* locus.

### Targeted transgene expression in human primary B cells

**Fig. 2:**
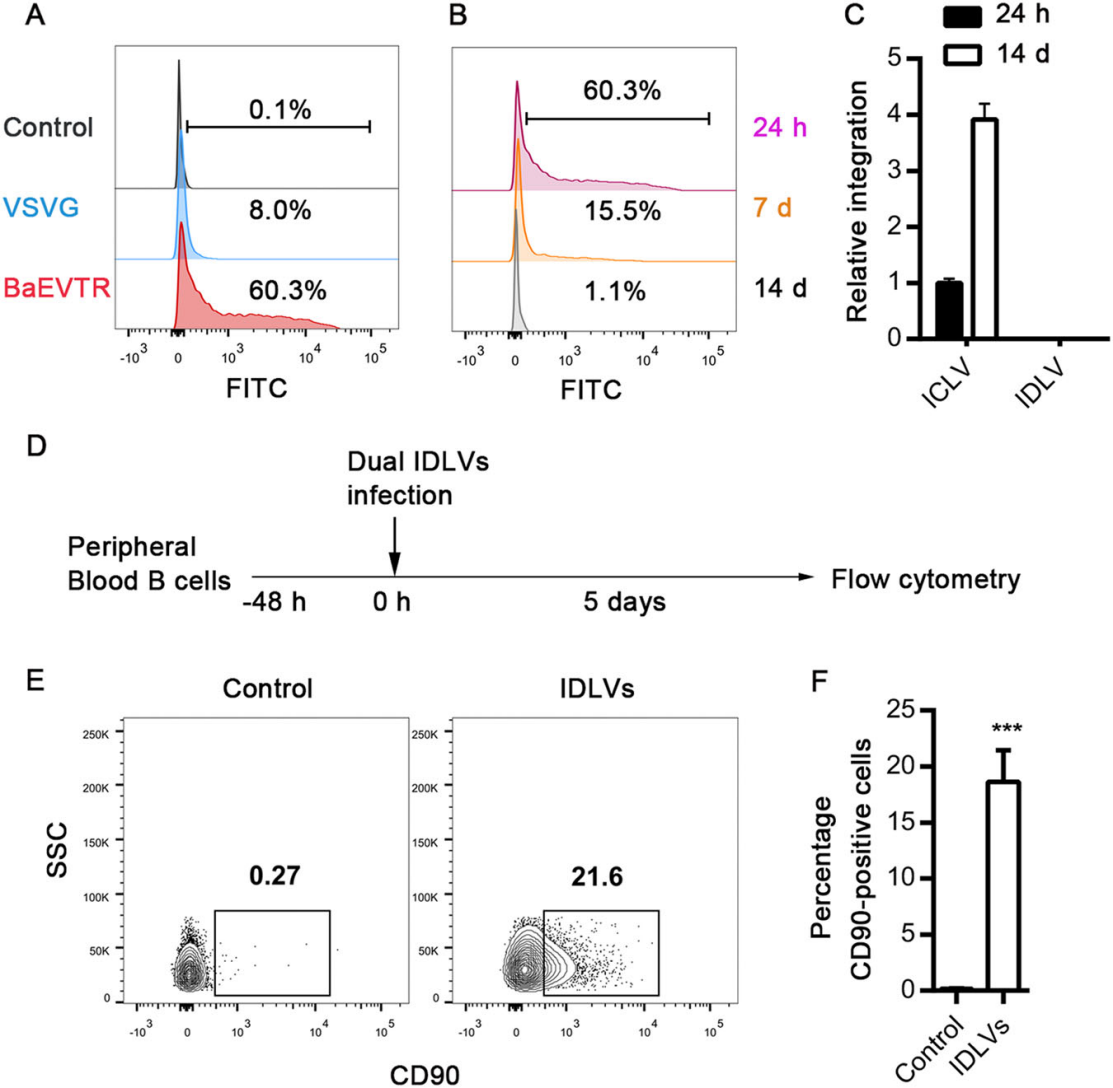
Efficient targeted integration of α-PD-1 mAb into human primary B cells. **a** Comparison of transduction rates between BaEVTR and VSVG pseudotyped IDLVs. An incubation of the freshly pre-stimulated B cells with BaEVTR or VSVG pseudotyped GFP-encoding IDLVs (MOI of 10, based on titering via flow cytometry for GFP expression) was conducted for 48 h at 37 °C, followed by FACS analysis for detection of GFP^+^ cells. Pre-stimulated B cells without transduction were used as the control. **b** A wave of transgene expression was observed at human primary B cells transduced with BaEVTR pseudotyped IDLV. GFP expression was estimated by FACS at 24 h, day 7 or day 14 post transduction. **c** Detection of BaEVTR pseudotyped ICLVs and IDLVs integration at 24 h or day 14 post transduction with a modified Alu-LTR nested–PCR protocol. Results are presented as mean ± SEM, *n* = 3. **d** Schematic representation of the human primary B cells engineering protocol by infection of dual-IDLVs. **e** Representative flow cytometric analysis for integrated CD90 expression 5 days post infection as indicated in **d**. Pre-stimulated B cells with only the donor IDLV were used as the control. **f** The relative knock-in efficiency in human primary B cells transduced with dual-IDLVs are shown. Data are from three independent experiments (means ± SEM), ***P < 0.001; two-tailed Student’s *t* test (**f**) was used.

As baboon retroviral envelope (BaEVTR) mediated a much higher transduction efficiency in human primary B cells^8^, we compared the transduction efficiency mediated by BaEVTR pseudotyped IDLVs with that by vesicular-stomatitis-virus-G protein (VSVG) pseudotyped IDLVs in human primary B cells. BaEVTR pseudotyped IDLVs achieved >60% infection efficiency, which was much higher than that achieved by VSVG pseudotyped IDLVs (Fig. 2a). We next investigated the persistence of BaEVTR pseudotyped IDLVs. The episomal vectors displayed a ~4-fold reduction in the number of GFP-positive cells observed at day 7 and were completely undetectable after 14 days (Fig. 2b). Analysis of vector DNA revealed that the expression of HIV-1 RRE also rapidly went down to a very low level at day 14 (Fig. S1). Further, via a modified Alu-long terminal repeat (LTR) nested polymerase chain reaction (PCR) assay^39^, we did not find any randomly integrated provirus in the genomic DNA (Fig. 2c). These results demonstrate that BaEVTR pseudotyped IDLV appears to be an efficient and safe tool for transient expression in B-lymphocytes. Subsequently, we engineered human primary B cells using BaEVTR pseudotyped IDLVs (Fig. 2d). Co-infection of Cas9/sgRNA-expressing IDLV with HMEJ donor–transferring IDLV resulted in ~20% knock-in efficiency in primary B-lymphocytes (Fig. 2e, f). It is notable that, in contrast to the IDLV delivery system, we found that gene insertion mediated by electroporation of programmable CRISPR-Cas9 ribonucleoprotein complexes (RNPs) along with the transduction of an AAV carrying an α-PD-1 HR template in human primary B cells was much less ineffective (Fig. S2).

**Fig. 3:**
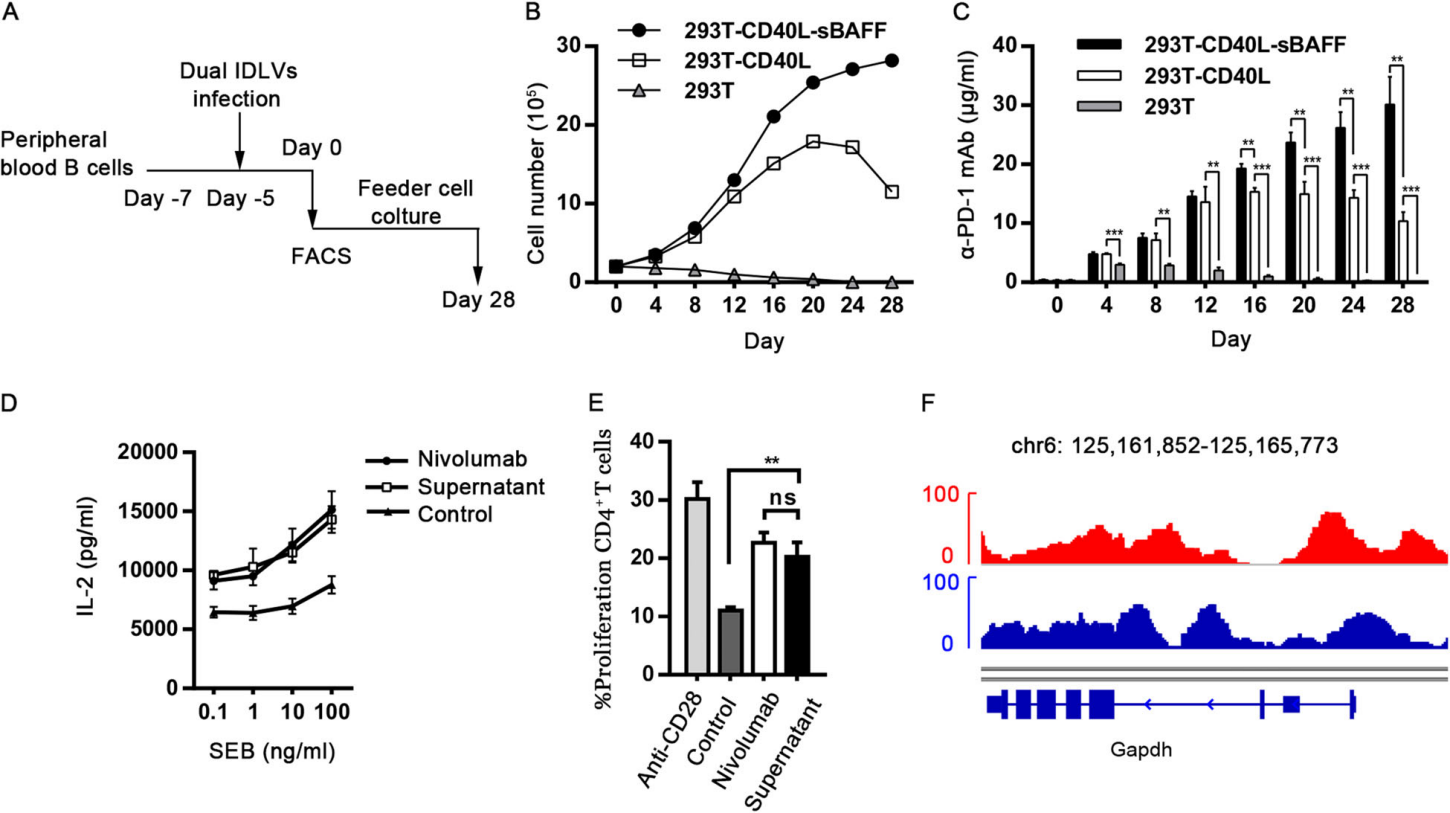
Functional α-PD-1 mAb secretion from engineered CD90-expressing B cells. **a** Schematic representation of sorting and expansion strategy of engineered CD90-expressing B cells. **b** Following FACS sorting at day 0 indicated in **a**, engineered CD90-expressing B cells were co-cultured with the irradiated 293T, 293T-CD40L, or 293T-CD40L-sBAFF feeder cells. The feeder cells were renewed every 4 days and the numbers of B cells were counted to indicate expansion patterns. **c** As performed in **a**, culture supernatants of gene-edited B cells co-cultured with feeder cells were collected at various time points, followed by ELISA for detecting α-PD-1 mAb concentration. Data are representative of three independent experiments. **d** PD-1 blockade-mediated T-cell stimulation assay was performed by SEB stimulation of PBMCs. 1 × 10^5^ PBMCs were stimulated with serial dilutions of SEB in the presence of nivolumab, culture supernatant of gene-edited B cells or untransduced B cells as a control. Supernatants were collected 3 days later and measured for IL-2 levels by ELISA. Nivolumab was used as a positive control. Representative data from three healthy donors are shown. **e** To conduct a T-cell proliferation assay mediated by PD-1 blockade, PBMCs from healthy donors were stimulated with anti-CD3 antibody and cultured in the presence of anti-CD28, nivolumab, culture supernatant of gene-edited B cells or untransduced B cells as a control for 3 days. The CFSE labeled CD4^+^ T cells were detected via flow cytometry. **f** Genome alignment tracks of the normalized ATAC-seq data showed the open chromatin for *GAPDH* locus in CD90^+^ engineered B cells cultured at day 28 (red). Pre-sitmulated B cells with only the donor IDLV were used as the control (blue). The results in panels **c**, **d**, and **e** are presented as mean ± SEM, *n* = 3. **P < 0.01, ***P < 0.001, ns, no significant difference; one-way ANOVA with Tukey’s post hoc tests (**c**–**e**) were used.

After IDLVs mediated production of gene-edited primary B cells, we sorted CD90-expressing B cells using flow cytometry and co-cultured them with the feeder cells (Fig. 3a). Consistent with our previous findings^40^, 293T-CD40L-sBAFF feeder cells supported a significant expansion of engineered B cells (Fig. 3b) with slight reduction of cell viability after co-culturing for 28 days (Fig. S3). The concentration of transgenic α-PD-1 mAb secreted in the culture supernatant increased along with the progressively increasing numbers of B cells (Fig. 3c). To assess the activity of transgenic α-PD-1 mAb in vitro, PD-1 blockade-mediated T-cell stimulation and proliferation assays were conducted, with nivolumab as the positive control. The α-PD-1 mAb secreted by engineered B cells enhanced the T-cell reactivity in a similar manner as nivolumab (Fig. 3d, e). Further, we detected the expression of endogenous *GAPDH* via an assay for transposase-accessible chromatin using sequencing (ATAC-seq) and found that the chromatin architecture at the *GAPDH* locus in human primary B cells was accessible. The accessibility did not alter 4 weeks after transgene cassette integration. These results indicate that the *GAPDH* locus is an excellent location for transgene integration and expression (Fig. 3f).

### The engineered B cells differentiated into LLPCs in vitro

**Fig. 4:**
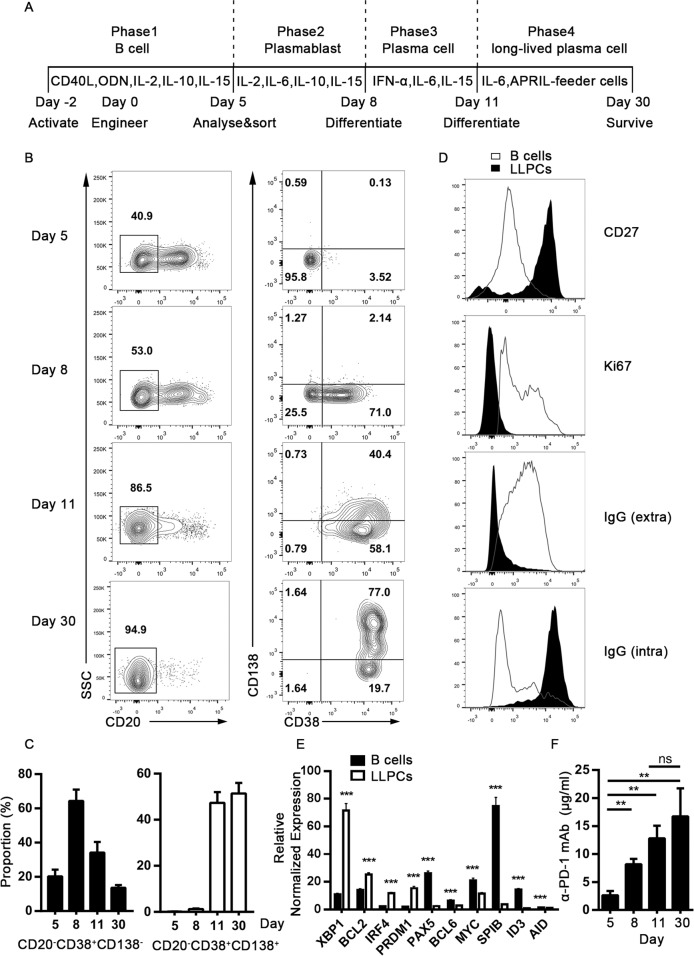
Differentiation of gene-edited human primary B cells into LLPCs in vitro. **a** Schematic representation of engineered B-cell differentiation into LLPCs in vitro using a multi-step cytokine culture system. **b** As described in **a**, engineered B cells differentiated into prePBs, PBs, PCs, and LLPCs respectively at indicated step. CD20, CD38, and CD138 staining were used for phenotype identification by FACS analysis at day 5, 8, 11, and 30 post gene-editing. **c** Proportion of plasmablasts and plasma cells at the end of the step are shown. **d** To confirm LLPCs phenotype, relative markers including CD27, ki67, extracellular IgG, and intracellular IgG were detected by FACS analysis. **e** Transcriptional signatures involved in LLPCs differentiation were tested by Quantitative real-time PCR. **f** The concentrations of α-PD-1 mAb in the supernatants were monitored at indicated time points during the differentiation process. Results are combined from three independent donors. The results in panels **c**, **e** and **f** are presented as mean ± SEM, *n* = 3. *P < 0.05, **P < 0.01, ***P < 0.001; two-tailed Student’s *t* test (**e**) and one-way ANOVA with Tukey’s post hoc test (**f**) were used.

To assess the ability of engineered B cells to differentiate in vitro, a multi-step cytokine culture system was used^41^ (Fig. 4a). At step one, site-specific gene-edited B cells were sorted using FACS and induced to differentiate into CD20^−^CD38^−^ pre-plasmablasts (prePBs) with MegaCD40L, CPG2006, IL-2, IL-10, and IL-15 in the culture medium. At step two, proliferating prePBs were cultured with IL-2, IL-6, IL-10, and IL-15 and allowed to differentiate into CD20^−^CD38^+^ PBs. At step three, PBs differentiated into the less proliferating CD20^−^CD38^+^CD138^+^ plasma cells in the presence of IL-6, IL-15, and IFN-α. At the last step, the PC culture was supported by IL-6 and the APRIL-secreting feeder cells for a prolonged period, resulting in the generation of CD20^−^CD38^+^CD138^+^ LLPCs (Fig. 4b, c). To verify the generation of LLPCs, specific markers of LLPCs, including CD27, ki67, extracellular IgG, and intracellular IgG, were detected via FACS analysis^41,42^. The newly generated LLPCs exhibited high expression levels of CD27, lacked positive ki67 staining and surface IgG expression, but had abundant IgG in the cytoplasm, indicating that they were immunoglobulin-producing cells without proliferation (Fig. 4d). In order to further validate the characteristics of the newly generated LLPCs, we quantified a series of transcriptional signatures^5,43,44^. These LLPCs expressed *PRDM1* and *IRF4* for PC transcription factors at a higher level but failed to express *PAX5*, *MYC*, *SPIB*, and *ID3*. Proliferation-related genes such as *BCL6* were expressed weakly but anti-apoptotic genes such as *BCL2* were overexpressed. They also exhibited high expression of *XBP1*, which is a master regulator of protein unfolding. Besides these, *AID* expression was downregulated (Fig. 4e). These gene expression patterns supported that these cells were LLPCs. During the process of differentiation, ELISA results indicated that the concentration of α-PD-1 mAb in the supernatant increased with PC maturation, although there was no significant statistical difference between PCs and LLPCs (Fig. 4f). In summary, our results indicated that site-specific integrated human primary B cells can be engineered into the typical LLPCs in vitro and maintain continuous transgenic antibody secretion.

### The engineered B cells differentiated into LLPCs upon transfer into immunodeficient NSG mice

**Fig. 5:**
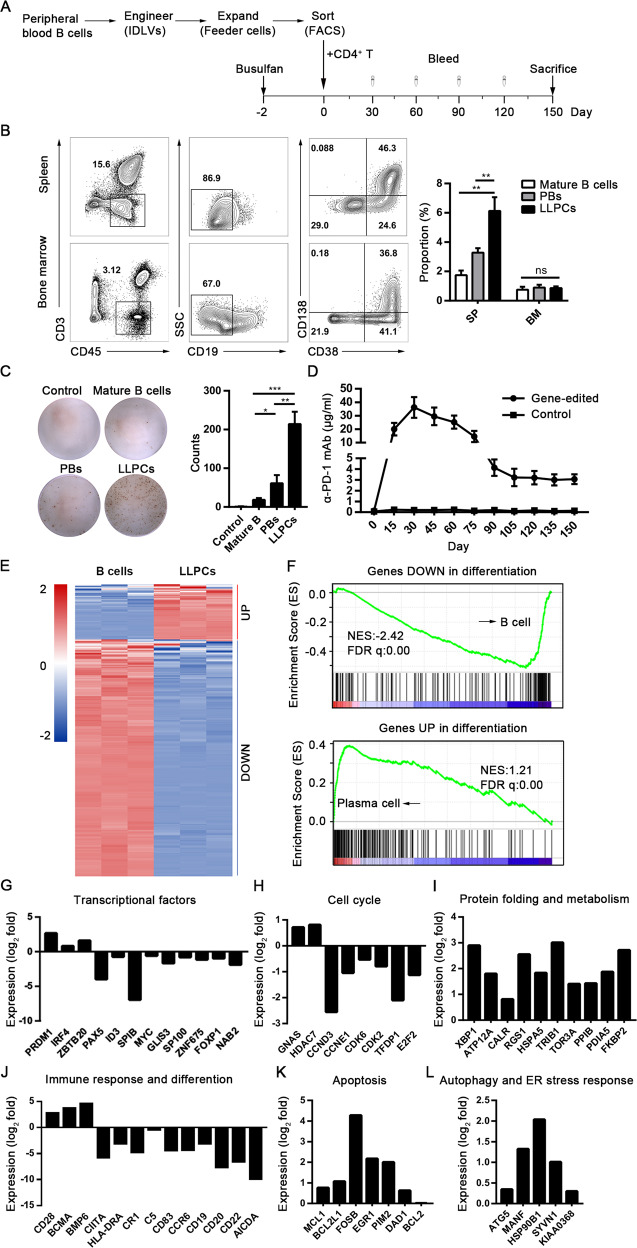
*G*eneration of α-PD-1 mAb secreting LLPCs from engineered human primary B cells upon transfer into NSG mice. **a** The strategy of LLPCs differentiation from engineered primary B cells upon transfer into NSG mice and 5 months of reconstitution. **b** Representative example of the spleen and bone marrow from humanized NSG mice. The identification of three subsets were shown as follows: mature B cells (CD19^+^), PBs (CD19^−^CD38^+^CD138^−^), and putative LLPCs (CD19^−^CD38^+^CD138^+^). The proportion of mature B cells, PBs and LLPCs are shown on the right panel. Results are the mean ± SEM from three individual mice. **c** The ELISpot assay results of the α-PD-1 mAb secreting subsets are shown from a representative experiment. The numbers of spot-forming cells/10^4^ cells are presented on the right panel as the mean number ±SEM from three separate experiments. *P < 0.05, **P < 0.01, ***P < 0.001, ns, no significant difference; one-way ANOVA with Tukey’s post hoc tests (**b**, **c**) were used. **d** The concentrations of α-PD-1 mAb were monitored in serum after the transfer of engineered B cells every 15 days for 5 months. Results from three independent donors were combined. Data are represented as mean ± SEM. **e** Selected “PC-related genes” are shown from three NSG mice sorted for B cells and LLPCs. Heatmaps showed the *z*-score normalized expression of the differentially expressed genes involved in the “PC-related gene” signature. RNA expression levels are indicated with a red/blue scale for high and low expression levels, respectively. **f** GSEA plots showed the enrichment genes of differentiation from B cells compared with plasma cells. The plot of running enrichment score (RES) is shown in green (top). Vertical bar (in black) in the middle indicate a gene within the differentiation gene set. The correlation of gene expression with subclusters is shown on the bottom. **g**–**l** Ratio of expression (log_2_ fold) in LLPCs to that in B cells for genes encoding transcription factors (**g**), cell cycle (**h**), protein folding and metabolism (**i**), immune response and B-cell differentiation (**j**), apoptosis (**k**), and autophagy and ER stress response (**l**). Data are pooled from three mice.

To study the development of engineered primary B cells into LLPCs, we adapted a humanized mouse model to generate human LLPCs using a method described previously^8^. Human primary B cells were co-cultured with 293T-CD40L-sBAFF and subsequently 293T-APRIL feeder cells for 7 days after the gene editing mediated by IDLVs as described above. Then, the engineered B cells sorted via FACS were adoptively transferred into NOD-Prkdc^scid^ Il2rg^null^ (NSG) mice. Rather than PBMCs that could induce xenograft-versus-host disease (xGVHD)^45^, primary CD4^+^ T lymphocytes, were co-injected to assist B-cell differentiation and homing to different hematopoietic tissues^8^. Blood samples were taken every 15 days and NSG mice were killed after 150 days of reconstitution. The cells of the spleen and bone marrow were isolated for FACS analysis (Fig. 5a). In this reconstituted model, the engineered B cells differentiated into several subsets residing both in the spleen and in bone marrow. They consisted of either CD19-positive mature B cells or CD19-negative subsets including PBs (CD19^−^CD38^+^CD138^−^) and the putative LLPCs (CD19^−^CD38^+^CD138^+^). The proportion of LLPCs was much higher than the two other subsets in the spleen (Fig. 5b). Further, we detected the antibody production from the three subsets by ELISpot assay and found that LLPCs secreted the largest amount of α-PD-1 mAb (Fig. 5c). Total α-PD-1 mAb levels in the serum detected by ELISA reached over 30 µg/ml at day 30 and then gradually declined, but still were maintained at ~3 µg/ml up to 150 days (Fig. 5d). Based on these results, it is obvious that the LLPCs differentiated from the engineered B-lymphocytes were mainly responsible for the long-term maintenance of α-PD-1 mAb level in the blood plasma.

As surface markers could be variable because of the PC subset heterogeneity^46,47^, LLPCs from the different hematopoietic tissues were sorted via flow cytometry and subjected to a global gene expression profiling analysis. To identify LLPC subsets, we defined a group of “PC-related genes” reported previously^48^. Gene expression analysis showed that the expression of 63 PC-related genes were upregulated and 271 PC-related genes were downregulated in LLPCs (Fig. 5e). Using gene set enrichment analysis (GSEA), we found that the B-cell gene sets were enriched within the downregulated genes, suggesting the repression of the B-cell gene expression by differentiation. Conversely, the plasma cell gene sets were enriched within the genes upregulated by differentiation (Fig. 5f). We further identified that the LLPC subset had the key transcriptional networks unique to LLPCs by deciphering the expression patterns of LLPC-specific genes (Fig. 5g–l). These LLPCs exhibited the intrinsic gene signature including the upregulation of *PRDM1*, *IRF4*, *XBP1*, *BCMA*, *CD28*, *ATG5*, and *BCL2*; and downregulation of *PAX5*, *ID3*, *HLA-DR*, *CD19*, and *E2F2*. All these genes are involved in transcription, cell cycle, protein folding, metabolism, immune response and differentiation, apoptosis, autophagy, and ER stress response: features specific for LLPCs^48–50^.

### The engineered B-cells secreting α-PD-1 mAb enhanced the antitumor activity of human T cells

**Fig. 6:**
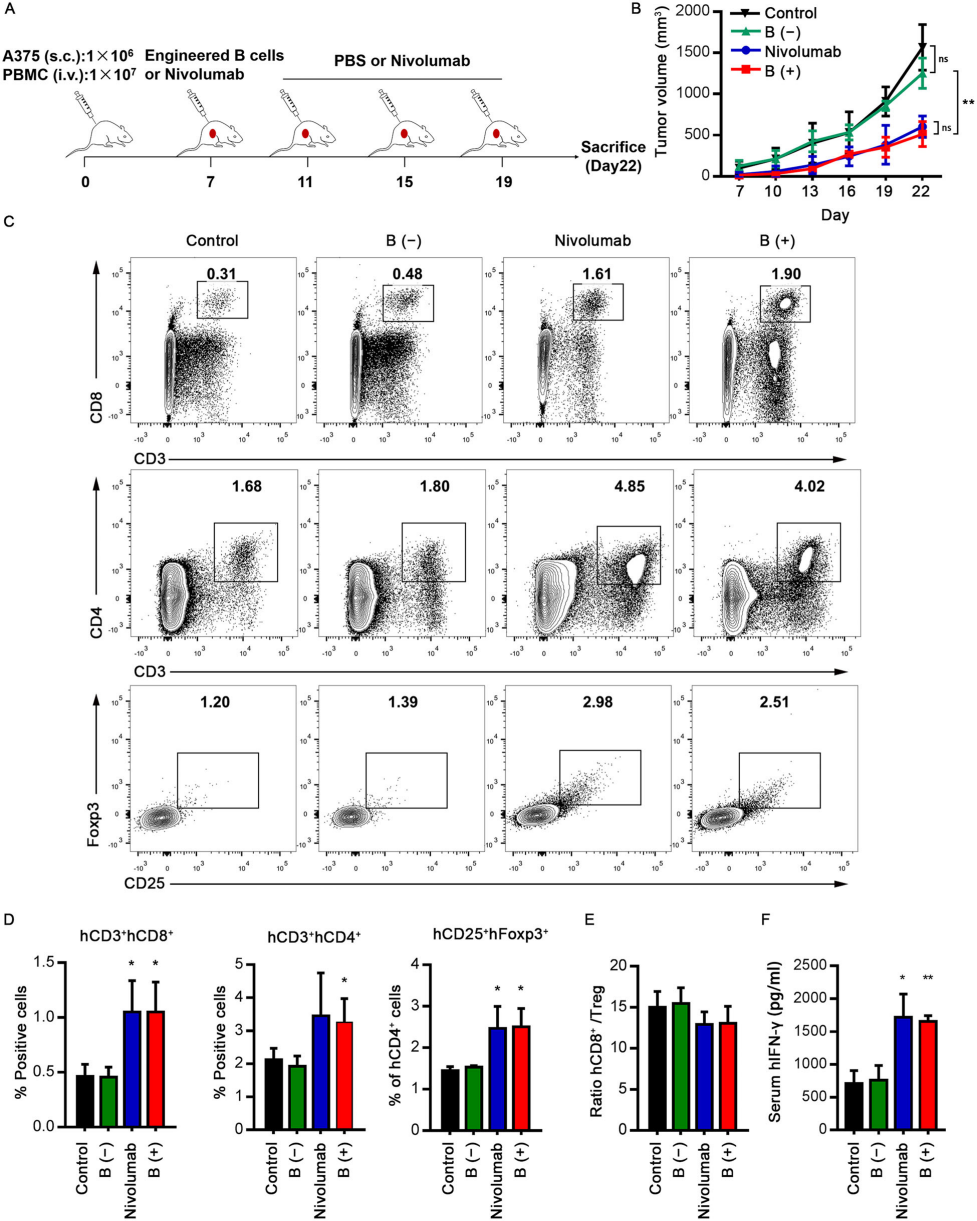
Enhanced antitumor activity of engineered human B cells via an antibody-mediated PD-1 blockade. **a** The strategy of detecting antitumor activity of engineered human B cells secreting α-PD-1 mAb in tumor-xenogenic humanized mice models. 1 × 10^6^ A375 melanoma cells were inoculated subcutaneously into the NSG mice, after which they were randomly sorted into four groups. Xenografted NSG mice were treated with engineered primary B cells, untransduced primary B cells, nivolumab, or isotype control. Nivolumab and isotype control were administrated three times a week. **b** Analysis of A375 melanoma xenografts growth. Tumor growth was evaluated at indicated time points (four mice in each group). **c**, **d** Representative flow cytometric analysis (**c**) and proportion (**d**) of tumor-infiltrating hCD4^+^ T cells, hCD8^+^ T cells and hCD25^+^ Foxp3^+^ Treg population. **e** The ratio of hCD8^+^ T cells/hTregs from data shown in **d**. **f** The serum hIFN-γ levels were measured by ELISA. The results in panels **b**, **d**–**f** are presented as mean ± SEM. Data shown are representative of three independent experiments. *P < 0.05, **P < 0.01, ns, no significant difference; one-way ANOVA with Tukey’s post hoc tests (**b**, **d**–**f**) were used.

It has been well known that α-PD-1 treatment significantly changes the clinical outcomes of melanoma patients. In order to evaluate whether human primary B cells engineered to secrete α-PD-1 mAb would induce a potent antitumor response, we used a xenografted tumor model in which human T-lymphocytes could be activated by injection of recombinant α-PD-1 antibody or engineered B-lymphocytes secreting α-PD-1 mAb. First, the human melanoma cell line A375 was subcutaneously inoculated into immunodeficient NSG mice concomitant with the adoptive transferred human PBMCs, which were HLA-matched with the A375 cells (Fig. S4). When tumors had grown to 3–5 mm in diameter, the mice were intravenously injected with either edited B cells, untreated B cells, nivolumab, or isotype control (Fig. 6a). The administration of one dose of edited B cells significantly abrogated the melanoma growth to comparable levels as repeated nivolumab injections (Fig. 6b). To address the mechanisms underlying the improved antitumor activity of administering edited B cells, tumor and blood samples were harvested at the end of the experiment. Treatment with edited B cells or nivolumab led to significant infiltrations of human CD4^+^ and CD8^+^ T lymphocytes in comparison with that of the control group (Fig. 6c). Analysis of another important immunosuppressive cell population, regulatory T lymphocytes (Tregs), showed that although a significant improvement in antitumor effect was observed when combined with edited B cells or nivolumab treatment, no significant change was observed in the ratio hCD8^+^ T cells/hTregs (Fig. 6d, e). In addition, significant secretion of serum hIFN-γ was observed in the edited B cell and nivolumab groups (Fig. 6f). Collectively, these results confirmed that the administration of edited B cells could inhibit human melanoma growth via an antibody-mediated PD-1 blockade. Similar results were obtained with a PD-L1 nearly negative human colon tumor cell line, which validated that the administration of gene-edited B cells had marked antitumor activity independent of high original PD-L1 expression (Fig. S5).

**Fig. 7:**
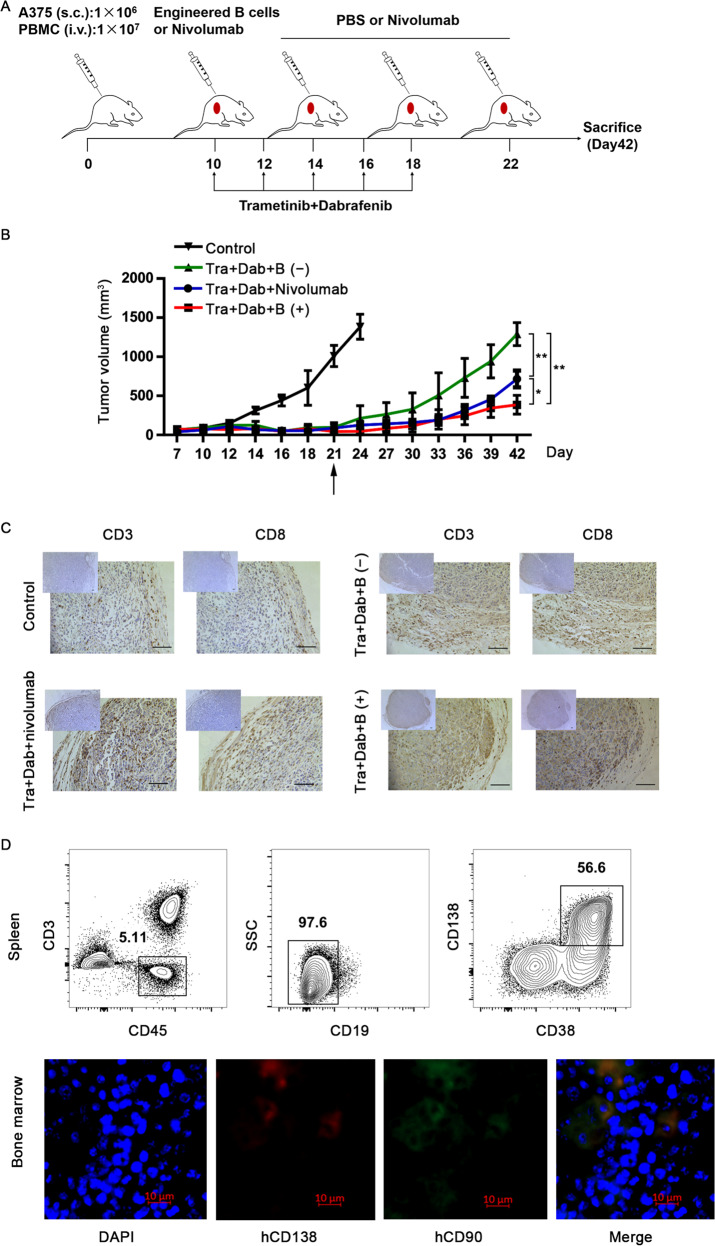
Long-term antitumor efficacy of engineered LLPCs in combinations with targeted inhibitors. **a** The strategy of investigating long-term efficacy of LLPCs combined with trametinib (Tra) and dabrafenib (Dab) treatment against human A375 melanoma. Mice treated only with vehicle (PBS, pH 7.0) were used as the control. Trametinib and dabrafenib were administrated every 2 days for 10 days. Nivolumab was given three times every week. **b** Tumor growth curves. Treatments began at day 10. Removing of inhibitors is marked by the black arrow. Representative graph of two repetitions of this experiment is shown. Data are presented as mean ± SEM. *P < 0.05, **P < 0.01; one-way ANOVA with Tukey’s post hoc test (**b**) was used. **c** The tumor infiltrating hCD3^+^ and hCD8^+^ lymphocytes. The melanoma biopsies were formalin fixed and processed for immunohistochemistry analysis at day 42. Anti-human CD3 antibody and anti-human CD8 antibody were used for primary staining (scale bar, 100 µm). **d** Representative flow cytometric analysis of LLPCs proportion in the spleen and representative immunofluorescence images indicating the engineered LLPCs population in the bone marrow. Human CD138 is shown in red, human CD90 in green, and DAPI-stained nuclei in blue. Scale bar, 10 μm.

Targeted inhibition of the mitogen-activated protein kinase (MAPK) pathway with selective BRAF and MEK inhibitors presents a new strategy to treat metastatic melanomas harboring *BRAF* mutations^51^. However, most patients develop drug resistance and eventually relapse^52^. A line of evidence has indicated that a synergistic effect occurs when immune checkpoint inhibitors and targeted therapies were used in preclinical modeling and clinical trials^53–56^. Such combination therapies may benefit patients by increasing the frequency of long-lasting antitumor responses. In order to explore the long-term treatment efficacy of engineered B cells, a melanoma-xenografted humanized mouse model was constructed to examine the treatment efficiency of dabrafenib plus trametinib, in combination with a PD-1 checkpoint inhibitor or engineered B cells. The mice were treated with a dabrafenib and trametinib combination via oral gavage once every 2 days for a total of 10 days. These mice were intravenously injected with edited B cells once, at the time of the first dose of targeted agents, or with nivolumab three times a week as the control (Fig. 7a). As expected, the dabrafenib and trametinib combinations showed marked antitumor activity although the tumor relapsed following drug withdrawal. However, the combination therapy with targeted agents plus nivolumab or engineered B cells significantly delayed tumor recurrence (Fig. 7b). The excised tumor tissues were analyzed at day 42. In the case of mice subjected to targeted agents and α-PD-1 blockade combination treatment, infiltrates of hCD3^+^ and hCD8^+^ lymphocytes were significantly more abundant than that in the case of the monotherapy (Fig. 7c). Furthermore, a high proportion of LLPCs was detected in the spleen in the combination therapy group at day 42 and LLPCs were detectable in the bone marrow, which suggest that α-PD-1 mAb secreted by LLPCs indeed significantly improved antitumor efficacy (Fig. 7d). Collectively, these results demonstrated that edited LLPCs, which persistently produced α-PD-1 mAb, maintained a long-term antitumor efficacy.

## Discussion

Adoptive cellular immunotherapy has developed gradually over the past two decades. T lymphocytes and natural killer (NK) cells, which are the main effector cells of the immune system, act as the main mediators of immunotherapy. However, more attention has been attributed to B-lymphocytes and plasma cells, with their role in cellular immunotherapy is beginning to be revealed. Strategies have been developed to reprogram human primary B cells to generate therapeutic antibodies and proteins^8,15,16,19,20^. Our study reported a novel approach to produce transgenic α-PD-1 mAb from human primary B cells using CRISPR/Cas9 technology. Especially, IDLVs pseudotyped with BaEVTR, supported the delivery of both Cas9/sgRNA and a large donor DNA template into human primary B cells, which resulted in high site-specific knock-in efficiency. We did not find any random integration of IDLVs, in contrast to a previous study showing that double stranded breaks (DSBs) generated by Cas9 nucleases may facilitate a 2- to 3-fold increase in IDLV integration^57^. It is notable that, previous studies have obtained transgenic antibody expression by introducing bnAb cassettes into human immunoglobulin heavy chain loci (*IgH*)^15,18–20^. However, such a strategy disrupts endogenous BCR expression and the resulting different heavy and light chain pairings may disrupt allelic exclusion and subsequently decrease transgenic antibody expression. Housekeeping genes offer good targets because it has been reported that active transcription enhances homologous recombination^58,59^. Our ATAC-sequencing data confirmed that *GAPDH* locus is chromatin accessible before and after transgene knock-in. Further, we have demonstrated that *GAPDH* is ubiquitously active, subsequently enabling CD90 expression and α-PD-1 mAb secretion upon correct integration. Therefore, our results support targeting of housekeeping genes for robust expression of therapeutic transgenes, and thereby avoiding endogenous gene disruption. Combining all the techniques together, we have demonstrated that gene-modified B-lymphocytes can differentiate into LLPCs both in vitro and in vivo in a humanized mouse model. LLPCs consistently secrete functional antibodies that significantly slow down tumor growth, indicating the potential clinical application of gene-edited LLPCs in adoptive cellular therapies.

It has been shown that LLPCs provide protective and durable immunity against a multitude of pathogens^49^. Unlike other immune cell subsets, the role of LLPCs in immunotherapy is less understood. LLPCs require organ-specific niches to provide them with survival factors, among which APRIL plays a key role^60^. The current study has demonstrated that a cytokine cocktail along with APRIL-feeder cell co-culture can induce gene-edited B cells to differentiate into LLPCs in vitro and sustain transgenic antibody secretion. After co-culture with 293T-CD40L-sBAFF and subsequently 293T-APRIL feeder cells, we analyzed the complexity of various B-cell differentiation subsets using a humanized mouse model. Transcriptome analysis further validated the characteristics of these LLPCs. Especially, we found that transcription factors maintaining the B-cell program including *PAX5* and *BCL6* were downregulated, while transcription factors including *PRDM1*, *XBP1*, and *IRF4* known to facilitate LLPC differentiation; *PRDM1* and *XBP1* required for antibody secretion; and *IRF4* required for PC survival were uniquely upregulated. In addition, some factors promoting LLPC maintenance; such as anti-apoptotic factors including *MCL1* and *BCL2*, and autophagy related genes such as *ATG5*, were increased. The activation of these regulatory networks promotes the expression of LLPC-specific features to produce large quantities of antibodies and survive for a long time. Therefore, the strategy and procedure to massively induce CRISPR/Cas9 engineered B cells into LLPCs as a novel platform for gene therapy merits being further optimized and standardized.

The immune checkpoint blockade, which has shown significant anti-cancer efficacy, requires repeated administration of blocking antibodies. Moreover, the function of recombinant antibodies may change due to post-translational modifications of their non-human or non-B lymphocytes origin. The B-lymphocyte or LLPC mediated systemic α-PD-1 mAb delivery may potentially overcome these drawbacks. In the xenografted melanoma mouse model, the effect of administered B cells engineered to express α-PD-1 mAb on abrogating melanoma growth was comparable to that of administration of multiple doses of nivolumab. Furthermore, when used in combination, engineered B cells and targeted inhibitors synergize to achieve a long-lasting antitumor activity. Secretion of the α-PD-1 mAb by LLPCs appears to be responsible for the treatment enhancement. It is important to note that, persistent secretion of α-PD-1 mAb by LLPCs might induce an excessively activated immune system, resulting in various side effects similar to the administration of recombinant α-PD-1 mAbs, including pneumonitis, hepatitis, myocarditis, and neurotoxicity^61^. To prevent these side effects, we have designed *CD90* in the cassette. Its expression on cell surface provides a marker for the selection, tracking, and ablation of engineered cells. When adverse events occur, the administration of α-CD90 mAb may eradicate the engineered LLPCs and prevent or minimize the risk of rare side effects. However, cell surface expression of CD90 has also been found in T cells, mesenchymal stem cells, and hematopoietic stem cells^62^. Treatment with α-CD90 mAb would inevitably lead to depletion of endogenous CD90^+^ stem cells. Besides, α-CD90 mAb is not commercially available pharmaceutical-grade monoclonal antibody specific for the epitope. Thus, CD90 is not a preferable safety marker upon gene-edited LLPC therapy. Epidermal growth factor receptor (EGFR) is not expressed by cells of the hematopoietic and lymphopoietic systems, which makes it as an attractive therapeutic molecule^63^. The truncated EGFR (EGFRt) could substitute CD90 as a selection marker, as well as a similar suicide gene^63–65^. The α-EGFR mAb, cetuximab, which has been widely used in clinical practice, could be used to easily eliminate the engineered LLPCs with EGFRt expression on the surface in vivo to avoid possible adverse effects. Adoptive gene-edited LLPC-based immunotherapy for long-term transgenic antibody expression may potentially eliminate the repeated injections in our experiments. However, gene-editing therapy involves substantial technical risks and ethical concerns^66^. The safety and ethics of first application of gene-edited LLPCs in clinical studies should be carefully considered. Notwithstanding the potential drawbacks of this approach, it is still a novel and important strategy for cancer immunotherapy.

Overall, the current proof-of-concept study has indicated that human primary B cells engineered to express α-PD-1 mAb with CRISPR/Cas9 technology mediated by IDLVs, followed by adoptively transferring them into the host where they differentiate into LLPCs and secrete therapeutic α-PD-1 mAb with an immune checkpoint blockade effect, is currently possible. We propose that this technology could be adapted not only for generating antibodies to eliminate various tumors or pathogen-infected cells, but also for correcting inherited or acquired gene deficiencies.

## Materials and methods

### Ethics statement

All mouse experiments were carried out in concert with the Sun Yat-sen University Laboratory Animal Center guidelines and were approved by the Institutional Animal Care and Use Committee of Sun Yat-sen University. The protocols were approved by the Institutional Animal Care and Use Committee at the Sun Yat-sen University. All efforts were made to avoid animal suffering.

### Cell lines

HEK293T and A375 cell lines were obtained from ATCC, and were cultured in Dulbecco’s modified Eagle medium (DMEM; Gibco, Invitrogen, Carlsbad, CA) supplemented with 10% fetal bovine serum (FBS; Gibco, Invitrogen, Carlsbad, CA) and 1% penicillin-streptomycin (Gibco) at 37 °C with 5% CO_2_. The A375 cell line was used for establishing the melanoma xenograft model. 293T-CD40L, 293T-CD40L-sBAFF, and 293T-APRIL cell lines were established according to our previous work^40^.

### Purification, culture, and activation of human primary B-lymphocytes

Peripheral blood mononuclear cells (PBMCs) derived from healthy human donors (Guangzhou Blood Center, Guangzhou) were isolated by Lymphocyte Separation Medium (TBD science). Then, the human primary B cells were negatively purified from PBMCs with an EasySep™ Human B Cell Isolation Kit (Stemcell) with a purity >90%. The medium for Human primary B cells culture was Iscove’s modified Dulbecco medium (IMDM, Invitrogen, Carlsbad, CA, USA) supplemented with 10% FBS, 1% penicillin-streptomycin, and 1% ITS Liquid Media Supplement (Sigma). Primary B-lymphocytes were activated with 10 μg/ml CpG oligonucleotide 2006/2219 (Invitrogen) and 50 ng/ml MegaCD40L (Enzo life science) in the presence of 20 U/ml hIL-2 (R&D Systems), 50 ng/ml hIL-10 (PeproTech), and 10 ng/ml hIL-15 (PeproTech). The cells were stimulated for 2 days in 12- or 24-well plates.

### Construction of Cas9/sgRNA and donor-encoding lentiviral vectors

The plasmid lentiCRISPR v2 encoding Cas9 and sgRNA was obtained from Addgene (Addgene#52961). SgRNA sequences targeting human *GAPDH* 3′ UTR were designed using the online tool CRISPR Design developed by Dr. Feng Zhang’s laboratory (https://zlab.bio/guide-design-resources). Pairs of sgRNA oligos for each targeting site were annealed and ligated into to the BsmBI restriction site upon (New England Biolabs) linearizing lentiCRISPR v2. Targeted sequences of sgRNAs used in this study are listed in Supplementary Table S1.

To construct the HR donor for the human *GAPDH* gene (Supplementary information, Data S1), 5′ and 3′ HAs (each 800 bp) were amplified from genomic DNA from human primary B cells and subcloned between the LTRs of lentiviral vector pCPPT-IRES-mStrawberry^67^. The P2A sequence and cloning sites were synthesized and used for amplifying the DNA fragment carrying nivolumab sequence from plasmid pLN539 (FuGW-G8p-antiPD1(HC)-P2A-antiPD1(LC)-1Pe; Addgene#105200). The T2A sequence was synthesized and used for generation of the T2A-CD90 fragment. The human CD90 CDS sequence was amplified by RT-PCR from the RNA of mesenchymal stem cells. After bridging P2A-α-PD-1 with T2A-CD90, the obtained DNA fragment was inserted into PacI and NotI sites between 5′ and 3′ HAs. The HMEJ donor was constructed by sandwiching donor DNA with 23 nt *GAPDH*-sgRNA target sequence (Supplementary information, Data S1).

The resulting fragments and linearized vectors were purified by Gel Extraction Kit (Omega). All plasmid constructs were extracted using the Endo-free Plasmid Mini Kit II (Omega) and verified by DNA sequencing.

### Transduction of recombinant integrase-deficient lentiviral particles

The integrase-deficient pseudo-viruses were generated by transfection of 3 × 10^6^ HEK293T cells with lentiviral vectors encoding Cas9/sgRNA or template donor (8.6 μg) and a packaging vector psPAX2-D64V (8.6 μg; Addgene#63586) using the calcium phosphate transfection method following the manufacturer’s instructions. For display of BaEVTR glycoprotein on IDLVs, 7 μg of plasmids coding for BaEVTR were co-transfected. For VSV-G-IDLVs production, 3 μg of VSV-G encoding plasmids (Addgene#12259) were co-transfected. Supernatants including vector particles were collected 48 h later and filtered through a 0.45-μm filter (Pall) to remove cell debris. The particles were concentrated by overnight centrifugation at 3000×*g* and 4 °C. Viral stocks were aliquoted and stored at −80 °C. Then, the activated B-lymphocytes were transduced with integrase-deficient lentiviral supernatants using retronectin-coated 48-well plates at the density of 1 × 10^5^/well, with polybrene (TR-1003-G, Sigma) at 8 μg/ml. Twelve hours later, the recombinant viruses were removed and B-lymphocytes were expanded in the conditioned medium as described above. Cells were collected for detection of knock-in efficiency 5 days post transduction.

### PCR detection of genomic integrations

Genomic DNA were extracted from cultured cells using Tissue DNA Kit (Omega) according to the manufacturer’s recommendations. Approximate 1 μg of genomic DNA was used for each PCR reaction and fragments were amplified by 2× Phanta Max Master Mix (Vazyme). Primers used for detection of genomic integration are listed in Supplementary Table S2.

### T7 endonuclease I assay

Genomic DNA was extracted as described above. SgRNA targeted region (~1300 bp) was amplified and purified using gel extraction kit (Omega) post electrophoresis. Then, 300 ng of purified PCR products were denatured and annealed using a thermocycler with the protocol as follows: 95 °C, 5 min; 95–85 °C at −2 °C/s; 85–25 °C at −0.1 °C/s and hold at 4 °C. The hybridized PCR products were digested with T7 endonuclease 1 (New England Biolabs) for 30 min at 37 °C. This reaction was stopped by adding 0.25 M EDTA and load the samples on the 1% agarose gel immediately. T7E1 cleavage efficiency was analyzed and quantified using ImageJ^38^. PCR primers are listed in Supplementary Table S2.

### Detection of virus integration

Genomic DNA was isolated and prepared for detection of virus integration at 24 h or day 14 post transduction. For vector copy numbers analysis, a modified Alu-LTR nested–PCR was performed^7^. Briefly, the first round of PCR allowed the amplification of up to 3 kb between an Alu sequence and a sequence downstream of 5′-LTR after the virus integrated. For the second round amplification, SYBR Ex-taq premix (Takara)-based quantitative PCR in a CFX96 Real-time PCR Detection System (Bio-Rad) was used to detect a 172-bp sequence of the proviral LTR.

To quantify integration of ICLVs (integrase-competent lentiviral vectors) and IDLVs, genomic DNA was digested with RNase A (Omega) and DpnI (New England Labs) overnight at 37 °C. Primers used to amplify vector DNA fragments are listed in Supplementary Table S2. Quantitative real-time PCR was performed and results were analyzed by CFX Manager (Bio-Rad). Human *ACTB* was measured as the endogenous control.

### Flow cytometry

Cells were washed and collected in PBS before FACS analysis. The single-cell suspensions were labeled for 20 min with various antibodies. Anti-human PE/cy7-CD90 (clone 5E10) and Fixable Viability Dye (eFluor™ 660) from eBioscience were used for knock-in efficiency analysis. For LLPCs phenotyping, the following antibodies were used: anti-human PERCP/cyanine 5.5-CD45 (clone HI30, Biolegend), PE-CD3 (clone OKT3, eBioscience), APC-CD19 (clone HIB19, eBioscience), APC-CD20 (clone 2H7, eBioscience), PE/cy7-CD38 (clone HIT2, Biolegend), Brilliant Violet 421™-CD138 (clone MI15, Biolegend). For identifying CD4^+^ T, CD8^+^ T and Treg cells, the following antibodies were used: anti-human PE/cy7-CD3 (clone UCHT1, eBioscience), PE-CD4 (clone OKT4, eBioscience), FITC-CD8 (clone HIT8a, QuantoBio), APC-CD25 (clone M-A251, Biolegend), and APC/cy7-Foxp3 (clone 236A/E7, eBioscience). For intracellular staining, cells were fixed and permeabilized using BD Cytofix/Cytoperm kit following recommendations of the manufacturer. The following antibodies were used for staining: anti-human PERCP-eFluor 710-CD27 (clone O323, eBioscience), FITC-IgG (clone G18–145, BD Horizon), and PE-Ki-67 (clone 20Raj1, eBioscience). Stained samples were acquired on a BD FACSAria and data were analyzed using FlowJo software (Tree Star, Ashland, OR).

### In vitro T-cell functional assays–Staphylococcal enterotoxin B stimulation of PBMCs and T-cell proliferation assay

In all, 1 × 10^5^ PBMCs from healthy human donors were cultured for 3 days with nivolumab (20 μg/ml, Opdivo, Bristol-Myers Squibb), supernatant of engineered human primary B cells or untransduced B cells together with serial dilutions of staphylococcal enterotoxin B (SEB; Toxin Technology). IL-2 levels in culture supernatants were measured with a Human IL-2 ELISA Kit (MultiSciences) and analyzed on GloMax® Discover (Promega).

In a T-cell proliferation assay, 2 × 10^5^ PBMCs from healthy donors were stimulated with soluble anti-CD3 antibody (1 ng/ml, eBioscience). PBMCs were cultured in the presence of anti-CD28 antibody (1 μg/ml, Stemcell), nivolumab (20 μg/ml), supernatant of engineered human primary B cells or untransduced B cells for 3 days. Cells were labeled with CFSE at the initiation of the assay to measure CD4^+^ T cells proliferation by FACS analysis.

### In vitro generation of human LLPCs

LLPCs were differentiated in vitro using a multi-step culture system as previously described^41^. All cultures were performed in IMDM supplemented with 10% FBS, 1% penicillin-streptomycin, and 1% ITS Liquid Media Supplement. At the first step, isolated PBMCs were activated as outlined above and expanded for 5 days with the same cytokine cocktail. At the second step, PBs were generated by seeding cells in medium with IL-2 (20 U/ml), IL-6 (50 ng/ml, PeproTech), IL-10 (50 ng/ml), and IL-15 (10 ng/ml) for 3 days. At the third step, PBs differentiated into early PCs by adding hIL-6 (50 ng/ml), hIL-15 (10 ng/ml), and IFN-α-2b (500 U/ml, Merck) for 3 days. At the last step, early PCs differentiated into LLPCs through co-culturing with irradiated 293T-APRIL feeder cells and adding hIL-6 (10 ng/ml). Fresh medium and cytokines were renewed twice a week and cultures were maintained until day 30.

### Enzyme-linked immunosorbent assay

Levels of human IFN-γ, IL-2, and α-PD-1 antibody in mouse serum samples or cell culture supernatants were measured by a commercial enzyme-linked immunosorbent assay kit (human IFN-γ ELISA set, Dakewei; human IL-2 ELISA kit, MultiSciences; human α-PD-1 antibody ELISA assay kit, Acro biosystem), according to the manufacturer’s instructions.

### Enzyme-linked immunosorbent spot

For enzyme-linked immunosorbent spot (ELISpot) assay, the PVDF plates were coated with PD-1 protein (2 μg/well, Sino Biological) overnight at 4 °C. Mature B cells, PBs, and LLPCs were sorted via flow cytometry and then added to PD-1 coated plates. Untransduced B cells were used as negative control. Plates were incubated for 16–20 h in a humidified atmosphere containing 5% CO_2_ at 37 °C. HRP conjugated anti-Human IgG-Fc Fragment (A80–104P, Bethyl) was used for detection of bound antibody. The ELISpot assays were then performed according to the manufacturer’s instructions. The plates were scanned by the S6 ultra immunoscan reader (Cellular Technology Ltd) and the number of α-PD-1 antibody positive cells was calculated by ImmunoSpot software (Version 5.1.34; Cellular Technology Ltd).

### Tissue processing and immunohistochemistry

Tumors were recovered and processed from mice at necropsy. Samples were stained according to standard procedures. Briefly, resected tumors were fixed with 4% formalin, embedded in paraffin, and applied to produce sections by Biopathology Institute Co., Ltd (Servicebio, China). Primary antibodies used for IHC staining were polyclonal rabbit anti-human CD3 mAb (17617-1-AP, proteintech) or rabbit anti-human CD8 mAb (EP1150Y, Abcam). Images of IHC sections were obtained using the microscope (Leica, DM6000B).

### Immunofluorescence

The bones resected from mice were decalcified in a decalcification solution (Merck). Then, the softened bones were immersed in Tissue-Tek O.C.T. Compound (4583, Sakura) and snap-frozen to produce cryosections. Primary antibodies used were as follows: mouse anti-human CD90 antibody (Clone 7E1B11, ab181469, Abcam) and rabbit anti-human Syndecan-1 antibody (Clone SP152, ab130405, Abcam). Alexa Fluor 488-conjugated donkey anti-mouse IgG H&L (ab150105, Abcam) and Alexa Fluor 647-conjugated donkey anti-rabbit IgG H&L (ab150075, Abcam) antibodies were used as secondary antibodies. DAPI (4′,6-diamidino-2-phenylindole; Thermo Fisher Scientific) was used for the staining of the nucleus. Fluorescent signals were detected using the laser scanning confocal microscope (ZEISS LSM 800).

### Genome-wide transcriptional profiling

RNA-seq was performed with two independent experiments per condition. For microarray analysis, total RNAs from mature B cells and LLPCs differentiated from the humanized mouse model were extracted by TRIzol Reagent (Thermo Fisher Scientific) according to the manufacturer’s instruction. The quality of RNA samples was evaluated by Nanodrop 2000 (Thermo Fisher). The RNA-Seq library was built with TruSeq Stranded mRNA Library Prep Kit (Illumina) and sequenced with Hiseq X Ten (Illumina) at BioMarker (Beijing, China) under the PE150 protocol. RNA-Seq reads were trimmed, filtered, and quality-controlled by FastQC (Babraham Institute) tool. Followed by calculating the reads per kilobase per million mapped reads (RPKM), the reads were aligned with human reference genome NCBI build 38 (GRCh38) by Hisat2^68^. Log_2_ fold-change (FC) ≥1 and FDR values <0.05 were used as the cut-off criterion. Through *z*-score normalization, the transcriptional profile data of differentially expressed genes were presented in a heatmap with MEV software (http://www.tm4.org/).

### ATAC-seq

To prepare ATAC-seq library, engineered α-PD-1 secreting B cells cultured at day 28 and activated primary B cells without transduction were sorted via flow cytometry. ATAC-seq was performed in biological duplicates following previous report^69^. Briefly, nucleus were isolated from 50,000 sorted cells per replicate using a solution of 10 mM Tris-HCl, 10 mM NaCl, 3 mM MgCl_2_, and 0.1% IGEPAL CA-630. Immediately following nuclei isolation, the transposition reaction was conducted using Nextera Tn5 transposase and TD buffer (Illumina) for 45 min at 37 °C. Transposed DNA fragments were purified using a Qiagen MinElute Kit, barcoded with dual indexes (Illumina Nextera), and PCR amplified using NEBnext High Fidelity 2× PCR master mix (New England Labs). The size distribution and molarity of the sequencing library were determined using an Agilent Bioanalyzer and KAPA quantitative RT-PCR (KAPA Biosystems). Sequencing was performed using an Illumina HiSeq X ten platform to acquire at least 50 M fragments per sample. Paired-end reads were mapped to the GRCh38 reference genome using Bowtie2. Only concordantly mapped pairs were kept for further analysis. Peak calling was performed using MACS v1.4 to identify areas of sequence tag enrichment. These peaks were displayed in the IGV genome browser and further processed for annotation and for differential open chromatin detection.

### Xenogenic mouse models

To assess the differentiation of gene-edited B cells into LLPCs, 4–6-week-old NSG (NOD-Prkdc^scid^IL2rg^tm1^/Bcgen, Beijing Biocytogen Co., Ltd) mice were intravenously (i.v.) injected with 5 × 10^6^ engineered CD90-positive B cells, which were co-cultured with 293T-CD40L-sBAFF and subsequently 293T-APRIL feeder cells for 7 days, along with 5 × 10^6^ autologous CD4^+^ T cells. Untransduced B cells along with autologous CD4^+^ T cells were transferred adoptively into the mice of the control group. Blood samples were taken every 15 days for antibody titration. Spleen and bone marrow cells were isolated for phenotypic analysis.

To observe antitumor activity of engineered B cells, 6–8-week-old female NSG mice were i.v. injected with 1 × 10^7^ PBMCs and inoculated subcutaneously (s.c.) into the right flank with 1 × 10^6^ A375 cells. When tumors growed to 3–5 mm in diameter, the mice were i.v. injected with either 5 × 10^6^ engineered CD90-positive B cells or untransduced B cells from the same PBMC donor once during the whole experimental cycle, or 200 μg nivolumab or isotype control antibody (IgG4, eBioscience) per mouse three times a week. Tumor size was measured using calipers every 3 days. To explore the long-term anti-tumor efficacy of LLPCs, 6–8-week-old female NSG mice were i.v. injected with 1 × 10^7^ PBMCs and inoculated s.c. with 1 × 10^6^ A375 cells. When tumors growed to 5 mm in diameter, mice were treated with 1 mg/kg trametinib (Tra, Selleck) and 30 mg/kg dabrafenib (Dab, Selleck) via oral gavage every 2 days for 10 days. Then, mice were i.v. injected with engineered B cells, untransduced B cells or nivolumab as described above. When we reached end-point mice were killed. Blood samples were harvested for human IFN-γ ELISA assay. Some tumor tissues were digested with collagenase type IV (2 mg/ml, Sigma) at 37 °C for 30 min and tumor-infiltrating lymphocytes were separated by centrifugation on a discontinuous Percoll gradient (Haoyang, China). Others were used for immunohistochemistry.

### Statistical analysis

Statistical tests were performed using Prism (GraphPad) software. All experimental data are presented as mean ± SEM. Two-tailed Student’s *t* tests and one-way ANOVA analysis of variance with Tukey’s post hoc tests were used. Data are considered statistically significant when *P* < 0.05, *P < 0.05; **P < 0.01; ***P < 0.001.

## Supplementary information

Supplementary Figure Legends

Supplementary Figure 1

Supplementary Figure 2

Supplementary Figure 3

Supplementary Figure 4

Supplementary Figure 5

Supplementary Materials
