# Supplementary material for: Engineering of α-PD-1 antibody-expressing long-lived plasma cells by CRISPR/Cas9-mediated targeted gene integration
Source: Cell Death Dis. 2020 Nov 12;11(11):973. doi: 10.1038/s41419-020-03187-1 (PMC7661525; doi:10.1038/s41419-020-03187-1)
Supplement: Supplementary file 1 — Supplementary Figure Legends [file 41419_2020_3187_MOESM1_ESM.docx]

**Supplementary figure 1. The persistence of BaEVTR pseudotyped ICLV and IDLV in infected HEK293T cells.** RRE expression of the vectors were detected by real-time PCR at day 1 and day 14 post transduction. Samples subjected to the qPCR assays were analyzed in triplicate. Data are presented as mean ± SEM. **P* < 0.05, ****P* < 0.001, ns, no significant difference; two-tailed Student’s t-test was used.

**Supplementary figure 2. Ineffective knock-in efficiency in human primary B cells at various MOIs with Cas9 protein/sgRNA (RNPs) electroporation and donor-AAV infection.** Pre-stimulated human primary B cells were treated with electroporation of programmable Cas9/sgRNA RNPs, and infected with AAV6 carrying *α-PD-1* template donor at MOIs of 10^4^, 10^5^, and 10^6^. The cells were harvested 5 days post infection for FACS analysis of CD90 expression. MOI indicates the amount of AAV added to cells. Data are representative of three independent experiments.

**Supplementary figure 3. Slightly reduced cell viability of** **engineered human primary B cells co-cultured with** **feeder cells.** Co-culture the engineered human primary B cells with 293T-CD40L-sBAFF feeder cells. Cell viability was assayed at indicated time points by trypan blue exclusion. Data are the mean percentage of viable cells ± SEM determined in three donors.

**Supplementary figure 4. HLA-compatibility of the PBMCs and the A375 cell line.** FACS analysis of HLA-A2 expression of the PBMCs and the A375 cell line before transferring into NSG mice.

**Supplementary figure 5. Engineered human B-cells secreting α-PD-1 mAb enhanced the antitumor activity of human T cells in colon carcinoma xenograft model.** (A) Comparison of PD-L1 expression levels between SW620 and A375 cell lines. (B) FACS analysis of HLA-A2 expression showed PBMCs were HLA-matched with the SW620 cell line. HLA-matched PBMCs were used for detecting tumor-antigen-specific immune response. (C) Analysis of SW620 colon carcinoma xenografts growth. Tumor growth was evaluated at indicated time points (at least 3 mice in each group). SW620 colon cancer cells at 4 × 10^6^ were inoculated subcutaneously into humanized NSG mice. NSG mice were treated with engineered primary B cells, untransduced primary B cells, nivolumab or isotype control. Mice were sacrificed at the end point. Spleen and serum of peripheral blood were collected for analysis in (D) and (E). (D) Representative proportion of tumor infiltrating hCD4^+^ T cells, hCD8^+^ T cells and hCD25^+^Foxp3^+^ Treg population. (E) The serum hIFNγ levels were measured by ELISA. The results in panels C, D and E are presented as mean ± SEM. Data shown are representative of three independent experiments. **P* < 0.05, ***P* < 0.01, ****P* < 0.001, ns, no significant difference; one-way ANOVA (C, D, E) with Tukey’s post hoc tests were used.
