# Supplementary figures and images for: Engineering of α-PD-1 antibody-expressing long-lived plasma cells by CRISPR/Cas9-mediated targeted gene integration
Source: Cell Death Dis. 2020 Nov 12;11(11):973. doi: 10.1038/s41419-020-03187-1 (PMC7661525; doi:10.1038/s41419-020-03187-1)

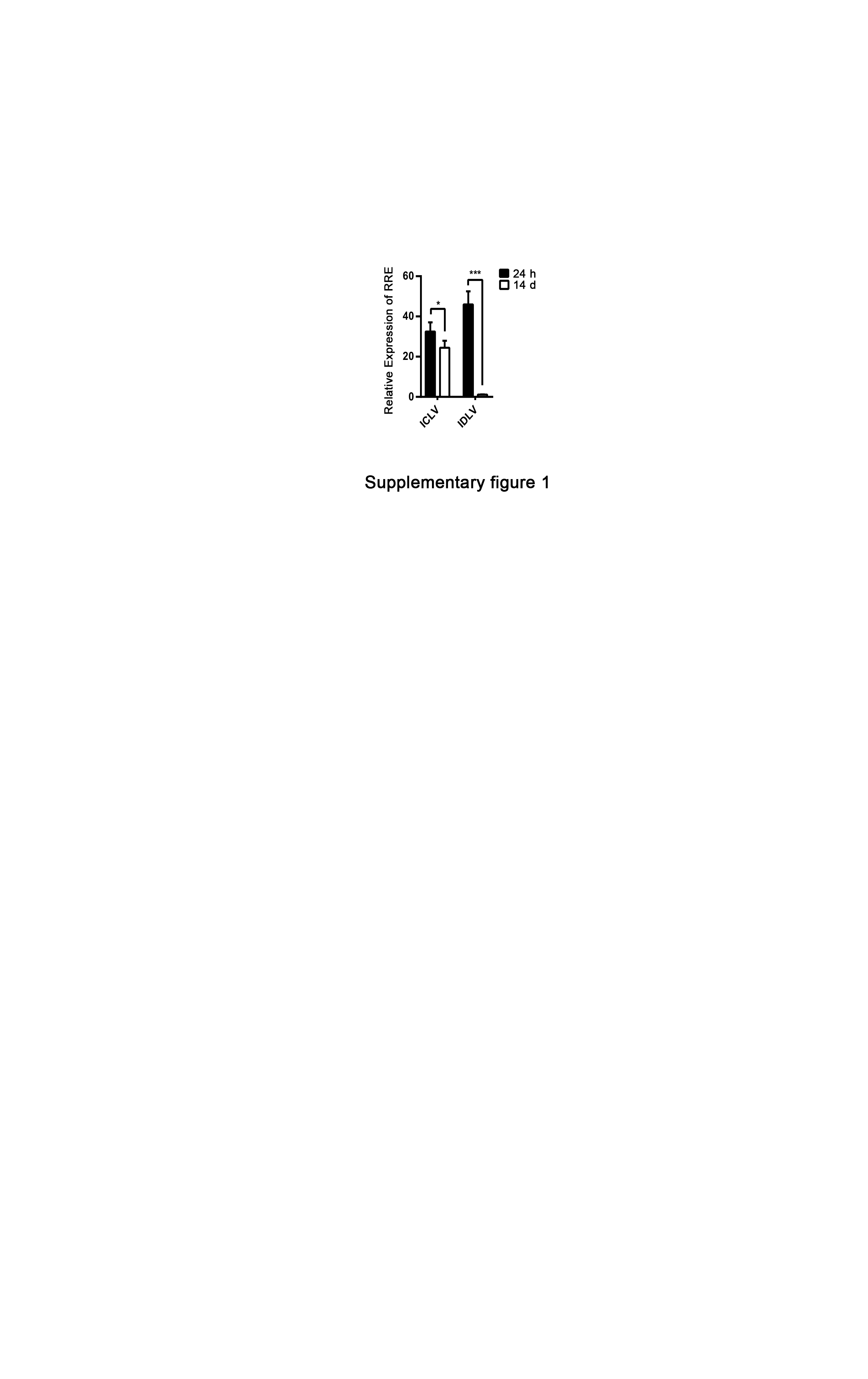

Supplement: Supplementary file 2 — Supplementary Figure 1 [file 41419_2020_3187_MOESM2_ESM.tif]

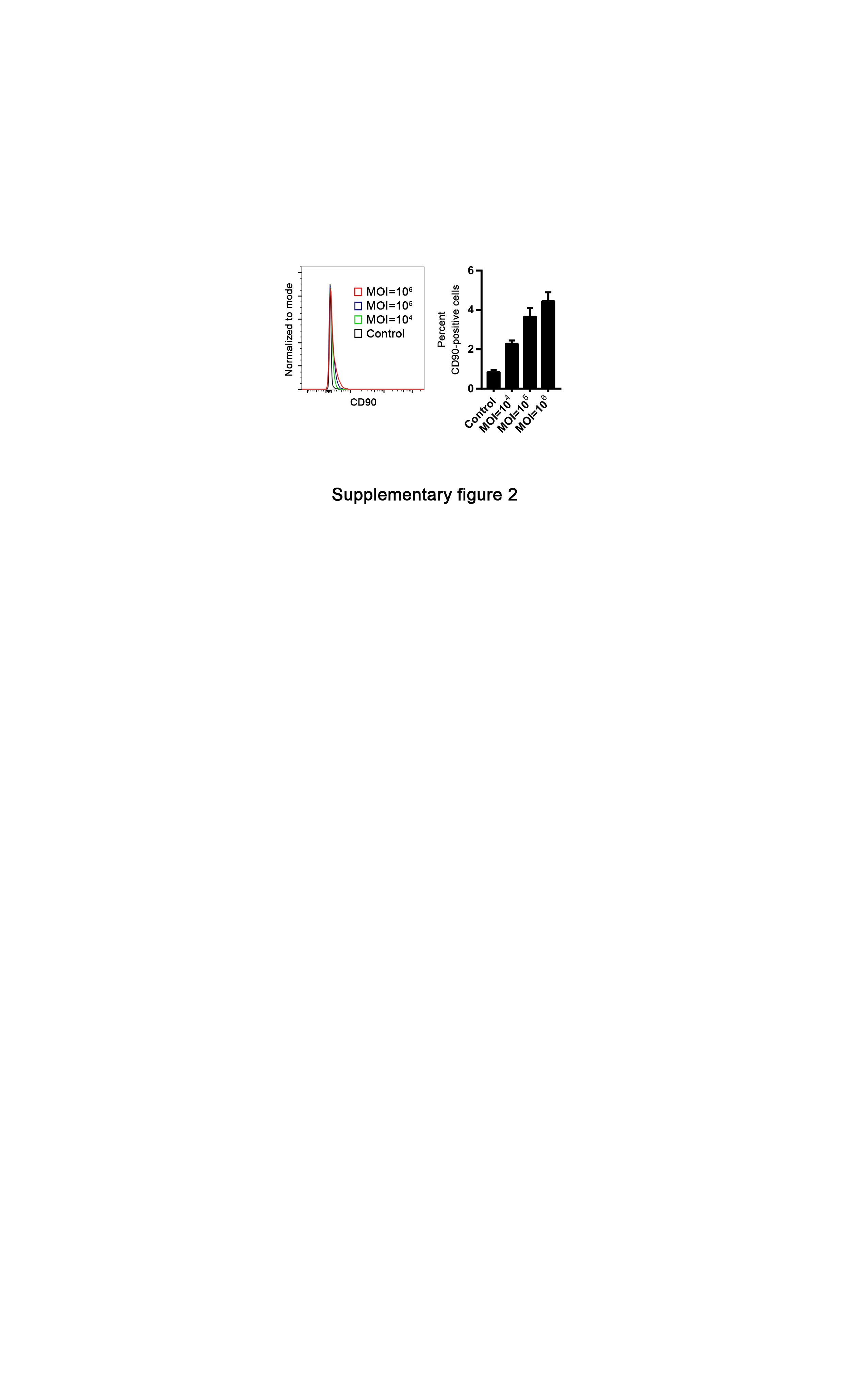

Supplement: Supplementary file 3 — Supplementary Figure 2 [file 41419_2020_3187_MOESM3_ESM.tif]

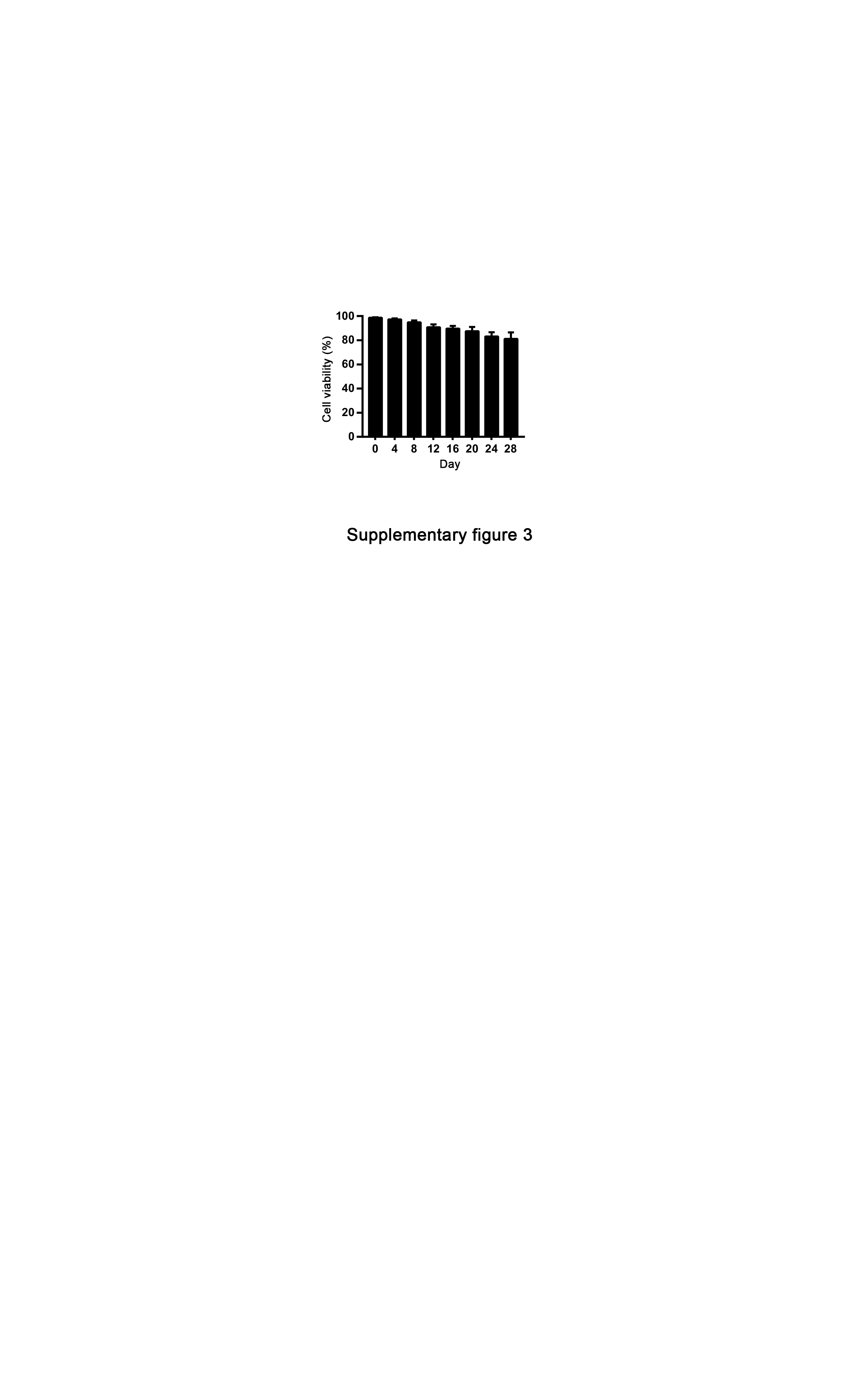

Supplement: Supplementary file 4 — Supplementary Figure 3 [file 41419_2020_3187_MOESM4_ESM.tif]

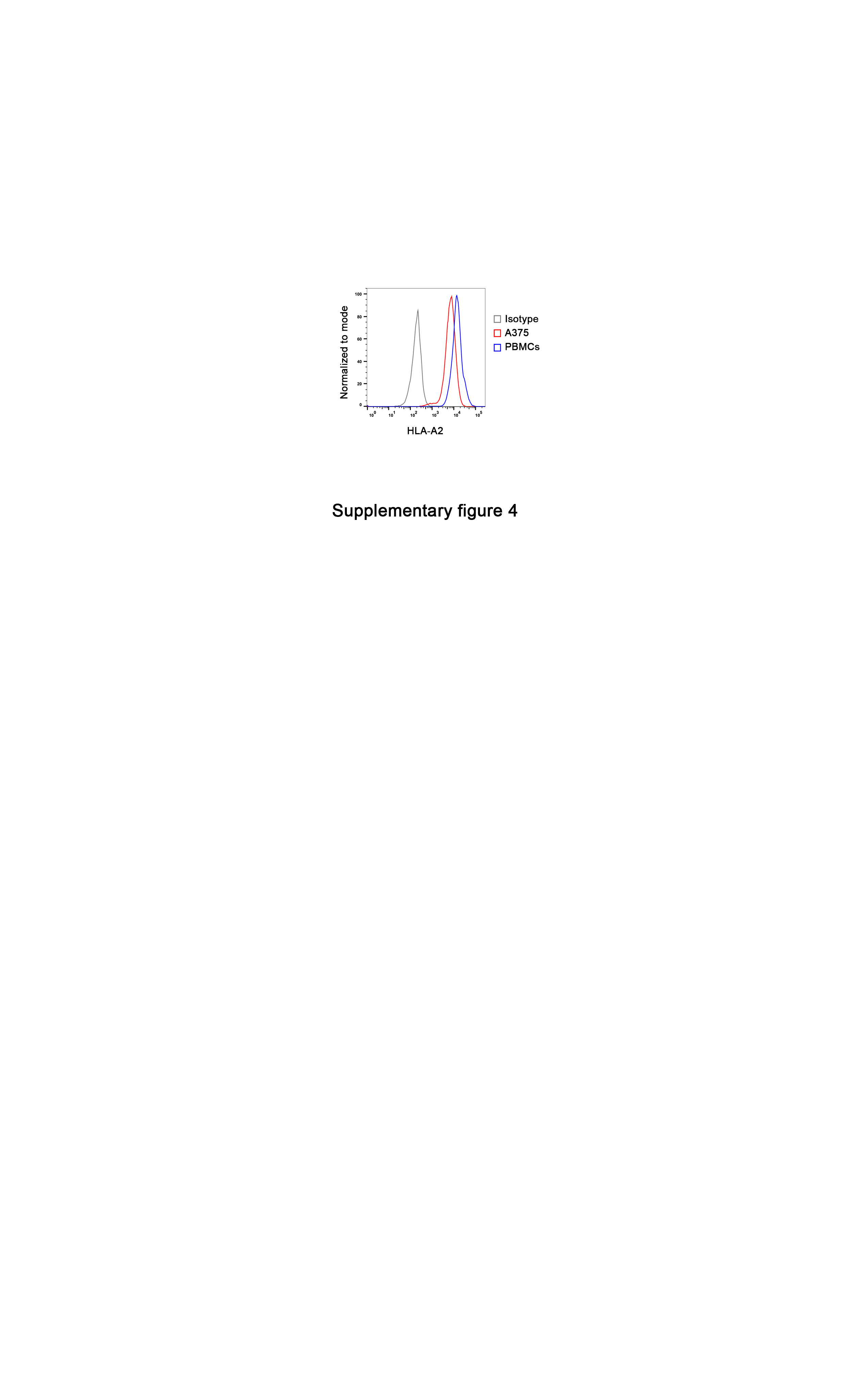

Supplement: Supplementary file 5 — Supplementary Figure 4 [file 41419_2020_3187_MOESM5_ESM.tif]

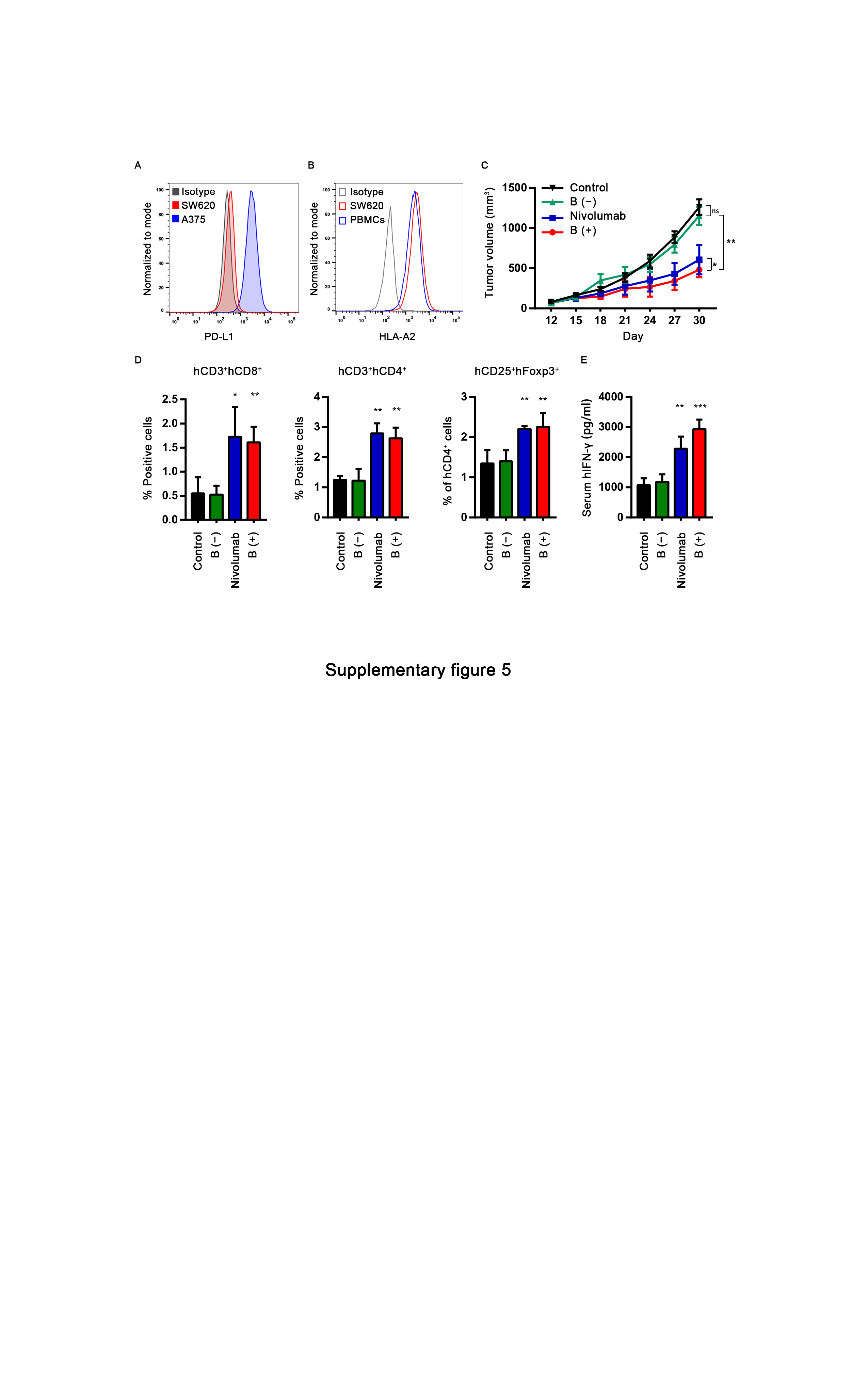

Supplement: Supplementary file 6 — Supplementary Figure 5 [file 41419_2020_3187_MOESM6_ESM.tif]
