# Supplementary material for: Engineering of α-PD-1 antibody-expressing long-lived plasma cells by CRISPR/Cas9-mediated targeted gene integration
Source: Cell Death Dis. 2020 Nov 12;11(11):973. doi: 10.1038/s41419-020-03187-1 (PMC7661525; doi:10.1038/s41419-020-03187-1)
Supplement: Supplementary file 7 — Supplementary Materials [file 41419_2020_3187_MOESM7_ESM.docx]

**Supplementary Table S1.** DNA sequences targeted by sgRNAs.

| sgRNA | Target sequences | PAM |
| --- | --- | --- |
| sg-1 | GGTGGACCTCATGGCCCACA | TGG |
| sg-2 | GAGAGAGACCCTCACTGCTG | GGG |
| sg-3 | AGCCCCAGCAAGAGCACAAG | AGG |

**Supplementary Table S2.** Primers used for T7E1 assay, knock-in detection and integrated virus detection.

| Primer | Sequences |
| --- | --- |
| *T7E1 assay* |  |
| GAPDH_T7E1-F | GACACGCTCCCCTGACTTGC |
| GAPDH_T7E1-R | CCTGTAGCACTCAAGACGTC |
| *Knock-in detection* |  |
| F1 | TCCCTCAATATGGTCCTGTCC |
| R1 | CTCTCTACGAGCTGCACCTG |
| F2 | GAGACAAACTGGTCAAGTGTG |
| R2 | CAGAGCCAGGGGTCTGGACCTA |
| *Integrated virus detection* |  |
| Alu-F | TCCCAGCTACTGGGGAGGCTGAGG |
| 5’LTR­_inner-F | TGGCTAACTAGGGAACCCACT |
| 5’LTR_downstream-R  RRE-F  RRE-R | CTTTCGCTTTCAAGTCCCTGTTC  TTGGGTTCTTGGGAGCAGCA  GATGCCCCAGACTGTGAGTTG |

**Supplementary Table S3.** Primers used for Real-Time PCR.

| Primer | Forward sequence | Reverse sequence |
| --- | --- | --- |
| XBP1 | 5’-CCGGAGCTGGGTATCTCAAAT-3’ | 5’-ATCCACAGTCACTGTAAGCATCCA-3’ |
| PRDM1 | 5’-AGCTTTCATCCCCTCGTACAAC-3’ | 5’-CGCTCAGGCCATTACAATTCAT-3’ |
| IRF4 | 5’-ACCCGCAGATGTCCATGAG-3’ | 5’-TGGCATCATGTAGTTGTGAACCT-3’ |
| BCL2 | 5’-CTTTGAGTTCGGTGGGGTCA-3’ | 5’-GAAATCAAACAGAGGCCGCA-3’ |
| PAX5 | 5’-CAGGACATGGAGGAGTGAATCA-3’ | 5’-ATGTCGCAGGGCCTGACA-3’ |
| BCL6 | 5’-TCCGTGCCCATGTGCTTA-3’ | 5’-GAGTCTGAAGGTGCCGGAAA-3’ |
| MYC | 5’-AGACAGATCAGCAACAACCGAAA-3’ | 5’-AGCTCCGTTTTAGCTCGTTCCT-3’ |
| SPIB | 5’-AGGGACTCGCAAGAAGCTG-3’ | 5’-GGTCTTGGCGTAGTTTCGGA-3’ |
| ID3 | 5’-AGCGCGTCATCGACTACATT-3’ | 5’-AGCTCCTTTTGTCGTTGGAGA-3’ |
| AID | 5’-CTTCAAAGATTATTTTTACTGCTGGAATAC-3’ | 5’-GATGCGCCGAAGCTGTCT-3’ |
| ACTB | 5’-CATGTACGTTGCTATCCAGGC-3’ | 5’-CTCCTTAATGTCACGCACGAT-3’ |

**Supplementary Information, Data S1.** Plasmid-based donor template sequences (5'-3').

Details of the plasmid based donor templates are as follows.

*GAPDH* HR donor (upper: homology arm; underline; linker)
TTGGTATCGTGGAAGGACTCATGGTATGAGAGCTGGGGAATGGGACTGAGGCTCCCACCTTTCTCATCCAAGACTGGCTCCTCCCTGCCGGGGCTGCGTGCAACCCTGGGGTTGGGGGTTCTGGGGACTGGCTTTCCCATAATTTCCTTTCAAGGTGGGGAGGGAGGTAGAGGGGTGATGTGGGGAGTACGCTGCAGGGCCTCACTCCTTTTGCAGACCACAGTCCATGCCATCACTGCCACCCAGAAGACTGTGGATGGCCCCTCCGGGAAACTGTGGCGTGATGGCCGCGGGGCTCTCCAGAACATCATCCCTGCCTCTACTGGCGCTGCCAAGGCTGTGGGCAAGGTCATCCCTGAGCTGAACGGGAAGCTCACTGGCATGGCCTTCCGTGTCCCCACTGCCAACGTGTCAGTGGTGGACCTGACCTGCCGTCTAGAAAAACCTGCCAAATATGATGACATCAAGAAGGTGGTGAAGCAGGCGTCGGAGGGCCCCCTCAAGGGCATCCTGGGCTACACTGAGCACCAGGTGGTCTCCTCTGACTTCAACAGCGACACCCACTCCTCCACCTTTGACGCTGGGGCTGGCATTGCCCTCAACGACCACTTTGTCAAGCTCATTTCCTGGTATGTGGCTGGGGCCAGAGACTGGCTCTTAAAAAGTGCAGGGTCTGGCGCCCTCTGGTGGCTGGCTCAGAAAAAGGGCCCTGACAACTCTTTTCATCTTCTAGGTATGACAACGAATTTGGCTACAGCAACAGGGTGGTGGACCTCATGGCCCACATGGCCTCCAAGGAGttaattaacgccactaacttctccctgttgaaacaagcaggggatgtcgaagagaatcccgggccagccaccatgggagttaaggtattgttcgcattgatttgcatagccgtggccgaagctcaggtgcagctcgtagagagcgggggaggtgtcgttcagccagggaggagtctgcggctggactgcaaagcctccgggattactttctcaaacagcgggatgcactgggtgaggcaggctcccggaaagggcctcgagtgggtggcggtaatatggtacgacgggagcaaacgctactacgcagacagtgtgaaaggaaggttcactatttctcgcgacaattcaaagaacaccctcttcctgcagatgaacagtctgcgggcagaagacaccgctgtctattactgtgccaccaatgacgattactggggccagggtaccctcgtgaccgtgtctagcgcctctaccaaaggaccatcagtttttcctcttgctccctgcagtcgctcaaccagcgagagcacagcagcgctgggatgtctggtgaaggattattttcctgagcccgtgactgtgagctggaattcaggcgccctgacgtccggcgtccatacattcccagcggtacttcaaagtagcgggttgtactctctctctagcgtggtaaccgtaccgagctcctccctggggacgaaaacgtatacatgtaatgtcgatcacaaaccatctaacacaaaagtggacaaacgcgttgagtccaagtatggccctccatgcccaccctgccccgcaccggagtttctgggcgggcccagtgtctttctgttcccaccgaagcctaaggacacgttgatgatctcaagaacacctgaagtcacctgcgtagtcgtggacgtttctcaggaggatcccgaggtccaattcaattggtacgttgatggagtggaggtccacaacgcaaagacaaagccgcgcgaagaacagttcaattctacttaccgcgttgttagcgtgctgactgtgctccaccaagactggctgaatggtaaggagtataagtgcaaggtgagcaataagggattgccatctagcatcgaaaagacaatatccaaagccaagggccaaccacgagagccacaagtgtacacgttgcctccctcacaagaagagatgaccaagaatcaagtgagcctcacttgcctggtcaagggattctacccttctgatatcgcagtggagtgggagtccaatggacagcccgagaacaactacaagacaacacccccagtgctggattccgacggctcattcttcttgtatagccggctgacagtggacaagagcaggtggcaggaaggaaatgtcttctcctgctccgtgatgcacgaggccctccacaaccactacactcagaaatctctctctctttcactcggtaaaggaagcggagctactaacttcagcctgctgaagcaggctggagacgtggaggagaaccctggacctggagttaaggtattgttcgcattgatttgcatagccgtggccgaagctgaaatagtgctgacccagtcccccgccaccctctctctctcacccggagagagggcaactctgtcctgcagggcctcccaatccgtaagcagctatctggcttggtaccagcaaaaacccggacaggcaccccggctgttgatctatgacgcctccaaccgagccactggcatccccgccagattctccggatctggatcagggactgattttaccctgactatcagttctctggaacctgaagattttgcagtttattattgtcagcaaagctctaactggccaagaacctttggccagggcacgaaggtcgagataaaaactgtcgcggccccaagcgtctttatctttccgccgtctgatgaacagctgaaatcagggaccgcctctgtggtgtgtctgctgaacaacttctacccaagggaagctaaggtgcaatggaaagtagacaatgccttgcagagcggcaactctcaggagagcgtaaccgaacaggatagtaaggattcaacttacagtctgagctctaccctgactctgagtaaggccgattacgagaagcataaagtctacgcatgcgaggtcacacatcaaggtctttcatctcccgtgacaaagtcctttaatcgaggggaatgcggatctggagagggcagaggaagtctgctaacatgcggtgacgtcgaggagaatcctggcccaatgaacctggccatcagcatcgctctcctgctaacagtcttgcaggtctcccgagggcagaaggtgaccagcctaacggcctgcctagtggaccagagccttcgtctggactgccgccatgagaataccagcagttcacccatccagtacgagttcagcctgacccgtgagacaaagaagcacgtgctctttggcactgtgggggtgcctgagcacacataccgctcccgaaccaacttcaccagcaaatacaacatgaaggtcctctacttatccgccttcactagcaaggacgagggcacctacacgtgtgcactccaccactctggccattccccacccatctcctcccagaacgtcacagtgctcagagacaaactggtcaagtgtgagggcatcagcctgctggctcagaacacctcgtggctgctgctgctcctgctctccctctccctcctccaggccacggatttcatgtccctgtgagcggccgcAAGATTAAGAGAGAGACCCTCACTGCTGGGGAGTCCCTGCCACACTCAGTCCCCCACCACACTGAATCTCCCCTCCTCACAGTTGCCATGTAGACCCCTTGAAGAGGGGAGGGGCCTAGGGAGCCGCACCTTGTCATGTACCATCAATAAAGTACCCTGTGCTCAACCAGTTACTTGTCCTGTCTTATTCTAGGGTCTGGGGCAGAGGGGAGGGAAGCTGGGCTTGTGTCAAGGTGAGACATTCTTGCTGGGGAGGGACCTGGTATGTTCTCCTCAGACTGAGGGTAGGGCCTCCAAACAGCCTTGCTTGCTTCGAGAACCATTTGCTTCCCGCTCAGACGTCTTGAGTGCTACAGGAAGCTGGCACCACTACTTCAGAGAACAAGGCCTTTTCCTCTCCTCGCTCCAGTCCTAGGCTATCTGCTGTTGGCCAAACATGGAAGAAGCTATTCTGTGGGCAGCCCCAGGGAGGCTGACAGGTGGAGGAAGTCAGGGCTCGCACTGGGCTCTGACGCTGACTGGTTAGTGGAGCTCAGCCTGGAGCTGAGCTGCAGCGGGCAATTCCAGCTTGGCCTCCGCAGCTGTGAGGTCTTGAGCACGTGCTCTATTGCTTTCTGTGCCCTCGTGTCTTATCTGAGGACATCGTGGCCAGCCCCTAAGGTCTTCAAGCAGGATTCATCTAGGTAAACCAAGTACCTAAAACCATGCCCAAGGCGGTAAGGACTATATAATGTTTAAAAATCGGTAAAAATGCCCACCTCGCATAGTTTTGAGGAAGATGAACTGAGATGTGTCAGGGT

*GAPDH* HMEJ donor (bold: sgRNA target sites; upper: homology arm; underline; linker)
**agccccagcaagagcacaagagg**TTGGTATCGTGGAAGGACTCATGGTATGAGAGCTGGGGAATGGGACTGAGGCTCCCACCTTTCTCATCCAAGACTGGCTCCTCCCTGCCGGGGCTGCGTGCAACCCTGGGGTTGGGGGTTCTGGGGACTGGCTTTCCCATAATTTCCTTTCAAGGTGGGGAGGGAGGTAGAGGGGTGATGTGGGGAGTACGCTGCAGGGCCTCACTCCTTTTGCAGACCACAGTCCATGCCATCACTGCCACCCAGAAGACTGTGGATGGCCCCTCCGGGAAACTGTGGCGTGATGGCCGCGGGGCTCTCCAGAACATCATCCCTGCCTCTACTGGCGCTGCCAAGGCTGTGGGCAAGGTCATCCCTGAGCTGAACGGGAAGCTCACTGGCATGGCCTTCCGTGTCCCCACTGCCAACGTGTCAGTGGTGGACCTGACCTGCCGTCTAGAAAAACCTGCCAAATATGATGACATCAAGAAGGTGGTGAAGCAGGCGTCGGAGGGCCCCCTCAAGGGCATCCTGGGCTACACTGAGCACCAGGTGGTCTCCTCTGACTTCAACAGCGACACCCACTCCTCCACCTTTGACGCTGGGGCTGGCATTGCCCTCAACGACCACTTTGTCAAGCTCATTTCCTGGTATGTGGCTGGGGCCAGAGACTGGCTCTTAAAAAGTGCAGGGTCTGGCGCCCTCTGGTGGCTGGCTCAGAAAAAGGGCCCTGACAACTCTTTTCATCTTCTAGGTATGACAACGAATTTGGCTACAGCAACAGGGTGGTGGACCTCATGGCCCACATGGCCTCCAAGGAGttaattaacgccactaacttctccctgttgaaacaagcaggggatgtcgaagagaatcccgggccagccaccatgggagttaaggtattgttcgcattgatttgcatagccgtggccgaagctcaggtgcagctcgtagagagcgggggaggtgtcgttcagccagggaggagtctgcggctggactgcaaagcctccgggattactttctcaaacagcgggatgcactgggtgaggcaggctcccggaaagggcctcgagtgggtggcggtaatatggtacgacgggagcaaacgctactacgcagacagtgtgaaaggaaggttcactatttctcgcgacaattcaaagaacaccctcttcctgcagatgaacagtctgcgggcagaagacaccgctgtctattactgtgccaccaatgacgattactggggccagggtaccctcgtgaccgtgtctagcgcctctaccaaaggaccatcagtttttcctcttgctccctgcagtcgctcaaccagcgagagcacagcagcgctgggatgtctggtgaaggattattttcctgagcccgtgactgtgagctggaattcaggcgccctgacgtccggcgtccatacattcccagcggtacttcaaagtagcgggttgtactctctctctagcgtggtaaccgtaccgagctcctccctggggacgaaaacgtatacatgtaatgtcgatcacaaaccatctaacacaaaagtggacaaacgcgttgagtccaagtatggccctccatgcccaccctgccccgcaccggagtttctgggcgggcccagtgtctttctgttcccaccgaagcctaaggacacgttgatgatctcaagaacacctgaagtcacctgcgtagtcgtggacgtttctcaggaggatcccgaggtccaattcaattggtacgttgatggagtggaggtccacaacgcaaagacaaagccgcgcgaagaacagttcaattctacttaccgcgttgttagcgtgctgactgtgctccaccaagactggctgaatggtaaggagtataagtgcaaggtgagcaataagggattgccatctagcatcgaaaagacaatatccaaagccaagggccaaccacgagagccacaagtgtacacgttgcctccctcacaagaagagatgaccaagaatcaagtgagcctcacttgcctggtcaagggattctacccttctgatatcgcagtggagtgggagtccaatggacagcccgagaacaactacaagacaacacccccagtgctggattccgacggctcattcttcttgtatagccggctgacagtggacaagagcaggtggcaggaaggaaatgtcttctcctgctccgtgatgcacgaggccctccacaaccactacactcagaaatctctctctctttcactcggtaaaggaagcggagctactaacttcagcctgctgaagcaggctggagacgtggaggagaaccctggacctggagttaaggtattgttcgcattgatttgcatagccgtggccgaagctgaaatagtgctgacccagtcccccgccaccctctctctctcacccggagagagggcaactctgtcctgcagggcctcccaatccgtaagcagctatctggcttggtaccagcaaaaacccggacaggcaccccggctgttgatctatgacgcctccaaccgagccactggcatccccgccagattctccggatctggatcagggactgattttaccctgactatcagttctctggaacctgaagattttgcagtttattattgtcagcaaagctctaactggccaagaacctttggccagggcacgaaggtcgagataaaaactgtcgcggccccaagcgtctttatctttccgccgtctgatgaacagctgaaatcagggaccgcctctgtggtgtgtctgctgaacaacttctacccaagggaagctaaggtgcaatggaaagtagacaatgccttgcagagcggcaactctcaggagagcgtaaccgaacaggatagtaaggattcaacttacagtctgagctctaccctgactctgagtaaggccgattacgagaagcataaagtctacgcatgcgaggtcacacatcaaggtctttcatctcccgtgacaaagtcctttaatcgaggggaatgcggatctggagagggcagaggaagtctgctaacatgcggtgacgtcgaggagaatcctggcccaatgaacctggccatcagcatcgctctcctgctaacagtcttgcaggtctcccgagggcagaaggtgaccagcctaacggcctgcctagtggaccagagccttcgtctggactgccgccatgagaataccagcagttcacccatccagtacgagttcagcctgacccgtgagacaaagaagcacgtgctctttggcactgtgggggtgcctgagcacacataccgctcccgaaccaacttcaccagcaaatacaacatgaaggtcctctacttatccgccttcactagcaaggacgagggcacctacacgtgtgcactccaccactctggccattccccacccatctcctcccagaacgtcacagtgctcagagacaaactggtcaagtgtgagggcatcagcctgctggctcagaacacctcgtggctgctgctgctcctgctctccctctccctcctccaggccacggatttcatgtccctgtgagcggccgcAAGATTAAGAGAGAGACCCTCACTGCTGGGGAGTCCCTGCCACACTCAGTCCCCCACCACACTGAATCTCCCCTCCTCACAGTTGCCATGTAGACCCCTTGAAGAGGGGAGGGGCCTAGGGAGCCGCACCTTGTCATGTACCATCAATAAAGTACCCTGTGCTCAACCAGTTACTTGTCCTGTCTTATTCTAGGGTCTGGGGCAGAGGGGAGGGAAGCTGGGCTTGTGTCAAGGTGAGACATTCTTGCTGGGGAGGGACCTGGTATGTTCTCCTCAGACTGAGGGTAGGGCCTCCAAACAGCCTTGCTTGCTTCGAGAACCATTTGCTTCCCGCTCAGACGTCTTGAGTGCTACAGGAAGCTGGCACCACTACTTCAGAGAACAAGGCCTTTTCCTCTCCTCGCTCCAGTCCTAGGCTATCTGCTGTTGGCCAAACATGGAAGAAGCTATTCTGTGGGCAGCCCCAGGGAGGCTGACAGGTGGAGGAAGTCAGGGCTCGCACTGGGCTCTGACGCTGACTGGTTAGTGGAGCTCAGCCTGGAGCTGAGCTGCAGCGGGCAATTCCAGCTTGGCCTCCGCAGCTGTGAGGTCTTGAGCACGTGCTCTATTGCTTTCTGTGCCCTCGTGTCTTATCTGAGGACATCGTGGCCAGCCCCTAAGGTCTTCAAGCAGGATTCATCTAGGTAAACCAAGTACCTAAAACCATGCCCAAGGCGGTAAGGACTATATAATGTTTAAAAATCGGTAAAAATGCCCACCTCGCATAGTTTTGAGGAAGATGAACTGAGATGTGTCAGGGT**cctcttgtgctcttgctggggct**
